# Alternative Pathway for 3-Cyanoalanine Assimilation in Pseudomonas pseudoalcaligenes CECT5344 under Noncyanotrophic Conditions

**DOI:** 10.1128/Spectrum.00777-21

**Published:** 2021-11-03

**Authors:** María D. Pérez, Alfonso Olaya-Abril, Purificación Cabello, Lara P. Sáez, M. Dolores Roldán, Conrado Moreno-Vivián, Víctor M. Luque-Almagro

**Affiliations:** a Departamento de Bioquímica y Biología Molecular, Edificio Severo Ochoa, Campus de Rabanales, Universidad de Córdoba, Córdoba, Spain; b Departamento de Botánica, Ecología y Fisiología Vegetal, Edificio Celestino Mutis, Campus de Rabanales, Universidad de Córdoba, Córdoba, Spain; University of Michigan-Ann Arbor

**Keywords:** *Pseudomonas pseudoalcaligenes*, 3-cyanoalanine, cyanide, LC-MS/MS quantitative proteomic analysis, nitrilase

## Abstract

3-Cyanoalanine and cyanohydrins are intermediate nitriles produced in cyanide degradation pathways in plants and bacteria. 3-Cyanoalanine is generated from cyanide by the 3-cyanoalanine synthase, an enzyme mainly characterized in cyanogenic plants. NIT4-type nitrilases use 3-cyanoalanine as a substrate, forming ammonium and aspartate. In some organisms, this enzyme also generates asparagine through an additional nitrile hydratase activity. The alkaliphilic bacterium Pseudomonas pseudoalcaligenes CECT5344 assimilates cyanide through an intermediate cyanohydrin, which is further converted into ammonium by the nitrilase NitC. This bacterium also contains three additional nitrilases, including Nit4. In this work, a proteomic analysis of *P. pseudoalcaligenes* CECT5344 cells grown with 3-cyanoalanine as the sole nitrogen source has revealed the overproduction of different proteins involved in nitrogen metabolism, including the nitrilase NitC. In contrast, the nitrilase Nit4 was not induced by 3-cyanoalanine, and it was only overproduced in cells grown with a cyanide-containing jewelry-manufacturing residue. Phenotypes of single and double mutant strains defective in *nit4* or/and *nitC* revealed the implication of the nitrilase NitC in the assimilation of 3-cyanoalanine and suggest that the 3-cyanoalanine assimilation pathway in *P. pseudoalcaligenes* CECT5344 depends on the presence or absence of cyanide. When cyanide is present, 3-cyanoalanine is assimilated via Nit4, but in the absence of cyanide, a novel pathway for 3-cyanoalanine assimilation, in which the nitrilase NitC uses the nitrile generated after deamination of the α-amino group from 3-cyanoalanine, is proposed.

**IMPORTANCE** Nitriles are organic cyanides with important industrial applications, but they are also found in nature. 3-Cyanoalanine is synthesized by plants and some bacteria to detoxify cyanide from endogenous or exogenous sources, but this nitrile may be also involved in other processes such as stress tolerance, nitrogen and sulfur metabolism, and signaling. The cyanide-degrading bacterium Pseudomonas pseudoalcaligenes CECT5344 grows with 3-cyanoalanine as the sole nitrogen source, but it does not use this nitrile as an intermediate in the cyanide assimilation pathway. In this work, a quantitative proteomic analysis by liquid chromatography-tandem mass spectrometry (LC-MS/MS) was performed to study, for the first time, the response to 3-cyanoalanine at the proteomic level. Proteomic data, together with phenotypes of different nitrilase-defective mutants of *P. pseudoalcaligenes* CECT5344, provide evidence that in the absence of cyanide, the nitrilase Nit4 is not involved in 3-cyanoalanine assimilation, and instead, the nitrilase NitC participates in a novel alternative 3-cyanoalanine assimilation pathway.

## INTRODUCTION

Nitriles are organic cyanides with important industrial applications in the synthesis of polymers and valuable chemicals (1, 2). However, the toxicity of nitriles and their main precursor, cyanide, may generate environmental concerns (3). Natural nitriles produced by plants, arthropods, and bacteria are usually associated with cyanide metabolism, having a defensive role against competitors or predators attributed to cyanide release by enzymatic hydrolysis (4, 5). Cyanohydrins are hydroxynitriles generated by some cyanide-degrading microorganisms, such as Pseudomonas pseudoalcaligenes CECT5344. In this bacterium, the cyanide assimilation pathway includes a nonenzymatic reaction between cyanide and oxaloacetate, forming a cyanohydrin that is further assimilated by the nitrilase NitC (6, 7).

3-Cyanoalanine (3-CNA) is a nitrile mainly found in cyanogenic plants and arthropods. The 3-CNA synthase (EC 4.4.1.9) is a pyridoxalphosphate-dependent enzyme that synthesizes 3-CNA from cysteine and cyanide through a substitution reaction. Although 3-CNA has mutagenic and carcinogenic effects, it is considerably less toxic than cyanide. For this reason, 3-CNA synthase is the principal mechanism to detoxify cyanide released from cyanogenic glycosides in cyanogenic plants (8). The presence of 3-CNA synthase in noncyanogenic plants also allows detoxification of cyanide produced by exogenous sources and during ethylene biosynthesis. Ethylene is a plant hormone formed from methionine through a pathway that generates *S*-adenosylmethionine and 1-aminocyclopropane-1-carboxylic acid (ACC) as intermediates. During the oxidation of ACC, catalyzed by the enzyme ACC oxidase, hydrogen cyanide (HCN) is liberated as a subproduct. Additional functions in stress tolerance, nitrogen and sulfur metabolism, plant development, and signaling have also been assigned to 3-CNA synthase in higher plants (8). The nitrilase NIT4, which is widely distributed in plants, enables complete cyanide detoxification. In *Arabidopsis*, NIT4 has dual nitrile hydratase/nitrilase activity, using 3-CNA to generate either asparagine or aspartate and ammonium (9).

Some bacteria may generate cyanide by the HCN synthase (10), and 3-CNA production has been described in Chromobacterium violaceum (11). Synthesis of 3-CNA by the 3-CNA synthase could also be a mechanism for exogenous cyanide detoxification in noncyanogenic microorganisms (12). In the cyanide-assimilating bacteria Bacillus megaterium and Enterobacter sp. strain 10-1, cyanide is assimilated through 3-CNA (13, 14). The synthesis of 3-CNA by marine cyanobacteria in cyanide-free media has been also described (15). In this case, 3-CNA acts as biocide against microalgae. To date, only two bacterial 3-CNA synthases, from Pseudomonas ovalis no. 111 (reclassified as Pseudomonas putida) and Bacillus stearothermophilus CN3 (reclassified as Geobacillus stearothermophilus), have been characterized. These enzymes are thermostable homodimers of 70 kDa with specificity for *O*-acetyl-l-serine (12, 16). The *G. stearothermophilus* 3-CNA synthase synthesizes both 3-CNA and cysteine (16).

In some bacteria, the ability to assimilate exogenous 3-CNA has been described. Thus, Pseudomonas sp. strain 13 has a 3-CNA-degrading activity that generates asparagine and aspartate through a NIT4-type nitrilase (17, 18), and homologs have also been identified in several plant growth-promoting bacteria (19, 20). The genome of the cyanide-degrading bacterium *P. pseudoalcaligenes* CECT5344 contains four nitrilase genes, the *nitC* (BN5_1632) gene, which is essential for cyanide assimilation (7), the BN5_1912 gene coding for the nitrilase Nit4, and the BN5_1925 and BN5_4427 genes encoding two additional nitrilases, Nit1 and Nit2, respectively (21). *P. pseudoalcaligenes* CECT5344 assimilates 3-CNA as the sole nitrogen source, but this compound is not an intermediate in the cyanide assimilation pathway (22). The nitrilase Nit4 from the strain CECT5344 uses 3-CNA as a substrate, but this nitrilase is induced by cyanide and not by 3-CNA (23, 24). Therefore, the mechanism by which this nitrile is assimilated in the absence of cyanide is unknown. In this work, a proteomic approach and a mutational analysis have been performed to determine the role of the *P. pseudoalcaligenes* CECT5344 nitrilases and other proteins induced by 3-CNA in the assimilation of this nitrile under noncyanotrophic conditions.

## RESULTS

### *P. pseudoalcaligenes* CECT5344 lacks a functional 3-CNA synthase.

In a previous work, it was reported that enzymatic activity for 3-CNA production from cyanide and cysteine, serine, *O*-acetyl-serine, or thiosulfate was undetectable in *P. pseudoalcaligenes* CECT5344 (22). Here, a search for a putative 3-CNA synthase enzyme was carried out using the amino acid sequence of the previously characterized 3-CNA synthase from P. putida (12). BLAST analysis revealed the existence in *P. pseudoalcaligenes* CECT5344 of four cysteine synthase homologs (CysM1, CysM3, CysK1, and CysK3) encoded by the genes BN5_1558 (38% identity), BN5_1910 (40% identity), BN5_1627 (84% identity), and BN5_1628 (89% identity). However, CysK1 and CysK3 proteins only showed a query coverage of 50% and 42%, with a length of 162 and 152 amino acid residues, respectively, about half the size of 3-CNA/cysteine synthases, corresponding to the N-terminal and C-terminal ends of these enzymes. A similar result, but with less identity, was obtained when the 3-CNA synthase amino acid sequence from *G. stearothermophilus* was used in the comparative analysis with the strain CECT5344 (16).

**FIG 1:**
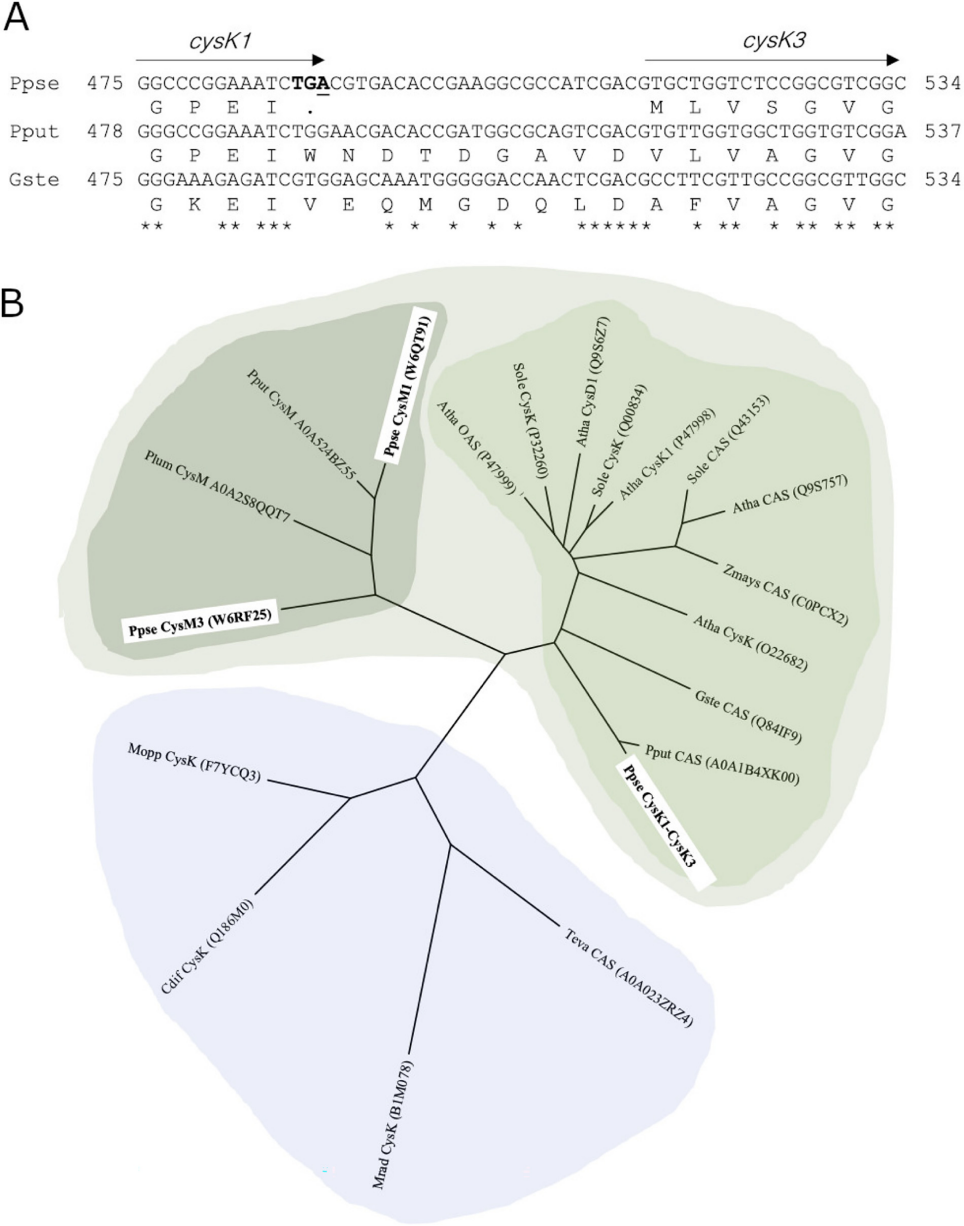
(A and B) Sequence alignment of the *P. pseudoalcaligenes* CECT5344 *cysK1*-*cysK3* intergenic region with nucleotide sequences of 3-CNA synthase genes of P. putida and *G. stearothermophilus* (A) and phylogenetic analysis of cysteine synthases and 3-CNA synthases (B). Conserved nucleotides are indicated by asterisks. The accession numbers of proteins correspond to the UniProt database. Ppse, Pseudomonas pseudoalcaligenes CECT5344; Pput, Pseudomonas putida; Gste, Geobacillus stearothermophilus; Plum, Photorhabdus luminescens; Mopp, Mesorhizobium opportunistum; Mrad; Methylobacterium radiotolerans; Cdif, Clostridioides difficile; Teva, Tetranychus evansi; Sole, Spinacia oleracea; Atha, Arabidopsis thaliana; Zmays, *Zea mays*.

When the nucleotide sequences were compared, the P. putida *casA* gene encoding the 3-CNA synthase (12) showed 100% query coverage and 82.6% identity with the joined sequence of BN5_1627 and BN5_1628 genes of *P. pseudoalcaligenes* CECT5344. When the 3-CNA synthase gene from *G. stearothermophilus* (16) was aligned against the genome sequence of the strain CECT5344, a lower identity (71%) was obtained. These nucleotide sequence alignments revealed that the TGA stop codon in the *P. pseudoalcaligenes* BN5_1627 gene corresponds to TGG (tryptophan) in P. putida or GTG (valine) in *G. stearothermophilus* (Fig. 1A). The BN5_1627 (*cysK1*) and BN5_1628 (*cysK3*) genes are in the same reading frame, separated by a 24-bp intergenic region that also showed high identity to the corresponding internal sequence of the P. putida *casA* gene (Fig. 1A). The artificially fused protein CysK1-CysK3 showed high identity (86 to 99%) with the full length (∼324 residues) of bacterial cysteine synthases. Like in *P. pseudoalcaligenes* CECT5344, two adjacent genes encoding the N- and C-terminal ends of putative cysteine synthases are found in Klebsiella michiganensis and Brucella abortus, indicating that this gene split event has also occurred in other bacteria.

A phylogenetic analysis of *P. pseudoalcaligenes* 3-CNA synthase homologs, including the fused protein CysK1-CysK3, was performed using protein sequences from bacteria, plants, and arthropods (Fig. 1B). Sequences were clustered into two main clades, one including several CysK (EC 2.8.5.1) from bacteria and a 3-CNA synthase with a bacterial origin described in mites and another clade that comprised two subgroups, one containing bacterial CysM (EC 2.5.1.113 and 2.5.1.144) sequences, including the CysM1 and CysM3 proteins of the strain CECT5344, and a second subgroup that comprises 3-CNA synthases from plants and the bacteria P. putida and *G. stearothermophilus*. This subgroup also included the fused protein CysK1-CysK3 of *P. pseudoalcaligenes* CECT5344 (Fig. 1B).

### Differential proteomic analysis of *P. pseudoalcaligenes* CECT5344 in response to 3-CNA.

To identify proteins involved in 3-CNA metabolism in *P. pseudoalcaligenes* CECT5344, a quantitative proteomic approach by liquid chromatography-tandem mass spectrometry (LC-MS/MS) was performed in cells grown with ammonium, 3-CNA, or a cyanide-containing jewelry-manufacturing residue as the sole nitrogen source, using three independent biological replicates for each condition (see Fig. S1 in the supplemental material). In the comparative study of 3-CNA versus ammonium, 150 proteins were significantly upregulated by the nitrile, with 85 proteins exclusive (not detected in ammonium) and 65 proteins overrepresented in 3-CNA. In addition, a total of 96 proteins were downregulated by 3-CNA, with 52 proteins overrepresented and 44 proteins exclusive of ammonium (Fig. S2, Table S1). The differential proteomic analysis between cyanide-containing residue and ammonium revealed that 154 proteins were upregulated by the residue (95 proteins exclusive and 59 overrepresented in the residue), whereas 140 proteins were downregulated by the residue (82 proteins exclusive and 58 proteins overrepresented in ammonium) (Fig. S2, Table S2). Finally, in the comparative analysis of 3-CNA versus residue, 141 proteins were upregulated by 3-CNA (91 proteins exclusive and 50 proteins overrepresented in 3-CNA), and 110 proteins were downregulated by 3-CNA (67 proteins exclusive and 43 proteins overrepresented in the residue) (Fig. S2, Table S3).

Cluster of Orthologous Genes (COG) database analysis revealed the functional categories of proteins up- or downregulated by 3-CNA (Fig. S3). Considering the Gene Ontology (GO) biological process categories (25), significantly changed GO groups for the proteins upregulated by 3-CNA were “nitrate assimilation,” “polyhydroxybutyrate biosynthetic process,” “polyamine transport,” and “nitrogen compound metabolic process.” In the case of the proteins downregulated by 3-CNA, the GO group “translation” was significantly enriched (Fig. S4).

In the differential analysis of 3-CNA versus ammonium, none of the proteins encoded by the cyanide resistance *cio* gene cluster (21, 26), except the histidinol-phosphate aminotransferase HisC, were found to be differentially expressed. On the contrary, the nitrilase NitC and other proteins encoded by the *nit1C* gene cluster required for cyanide assimilation (6, 7) were significantly induced by 3-CNA (Table 1). However, most proteins encoded by these *cio* and *nit1C* gene clusters were found to be overexpressed in the cyanide-containing residue (Table 1), as reported in previous studies (27). *P. pseudoalcaligenes* CECT5344 contains four nitrilases (21), which are grouped phylogenetically into different branches of two main clades (Fig. S5). The nitrilases NitC, Nit1, and Nit2 were found to be upregulated by 3-CNA compared to ammonium. In contrast, the nitrilase Nit4 was not induced by 3-CNA, although it was found to be overexpressed, like NitC and Nit1, in cells grown with the cyanide-containing residue (Table 1). The cyanase CynS required for cyanate assimilation (28, 29) was overproduced in cells grown with the jewelry residue, but it was not induced by 3-CNA (Table 1).

**TABLE 1:** Relevant proteins upregulated by 3-CNA and/or the jewelry residue in *P. pseudoalcaligenes* CECT5344

UniProt ID	Gene locus	Protein name	Fold change^*a*^		
			3CNA/NH_4_^+^	Residue/NH_4_^+^	3CNA/residue
Proteins encoded by the *cio* gene cluster (cyanide resistance)					
W6RF17	BN5_1900	Sulfite reductase hemoprotein β-component (CysI3)		R	
W6QU90	BN5_1901	Uncharacterized protein (CioC)		R	R
W6R254	BN5_1902	Terminal oxidase subunit I (CioA)		R	R
W6QWX6	BN5_1904	Phosphoserine aminotransferase (SerC)		R	R
W6RF21	BN5_1905	Histidinol-phosphate aminotransferase (HisC)	CNA	R	–243.78
W6QU95	BN5_1906	Acetylornithine aminotransferase (ArgD)		R	R
W6R260	BN5_1907	4-Hydroxy-tetrahydrodipicolinate synthase (DapA)		155.94	–139.07
W6QWY1	BN5_1909	Methylenetetrahydrofolate reductase (MetF)		R	R
W6RF25	BN5_1910	Cysteine synthase (CysM3)		R	R
W6QUA1	BN5_1911	NADP-dependent malic enzyme (MaeB)			–322.78
W6R265	BN5_1912	Nitrilase (Nit4)		147.93	–53.39
Proteins encoded by the *nit1C* gene cluster (cyanide assimilation)					
H9N5E0	BN5_1630	Sigma-54-dependent transcriptional regulator (NitA)	CNA		CNA
H9N5E2	BN5_1631	Uncharacterized protein (NitB)			–24.63
H9N5E1	BN5_1632	Nitrilase (NitC)	CNA	R	–70.60
H9N5E3	BN5_1633	Radical SAM domain-containing protein (NitD)		R	R
H9N5E4	BN5_1634	Acetyltransferase (NitE)		R	R
H9N5E5	BN5_1635	AIR synthase related protein domain protein (NitF)	CNA	R	–70.09
H9N5D8	BN5_1637	FAD-dependent oxidoreductase (NitH)	CNA	R	–11.91
Proteins encoded by the *cyn* gene cluster (cyanate assimilation)					
W6QRB2	BN5_0438	Fis family transcriptional regulator (CynF)	CNA		CNA
W6QST4	BN5_0439	ABC-type transporter periplasmic protein (CynA)	CNA	R	–190.04
W6RB18	BN5_0440	ABC transporter inner membrane subunit (CynB)		R	R
W6QQ36	BN5_0441	ABC transporter/ATPase component protein (CynD)		R	R
W6QY14	BN5_0442	Cyanase (CynS)		R	R
Other nitrilases					
W6RF39	BN5_1925	Aliphatic nitrilase (Nit1)	CNA	R	
W6R989	BN5_4427	Bifunctional nitrilase/nitrile hydratase NIT4B (Nit2)	5.30		5.63
Other relevant proteins					
W6QXC5	BN5_0151	Flavin monoamine oxidase	18.90	5.94	3.18
W6RAL1	BN5_0265	Putrescine transporter periplasmic protein	2.18		
W6QPL1	BN5_0266	Putrescine transport system substrate-binding protein	2.21		
W6QXK5	BN5_0267	Polyamine-transporting ATPase	2.38		2.56
W6QSH6	BN5_0329	Glutamate synthase	CNA		CNA
W6QXQ0	BN5_0332	N-carbamoyl-l-amino acid amidohydrolase	CNA	R	2.48
W6QSM0	BN5_0374	Substrate-binding ABC-type gly betaine transporter	2.02	2.28	
W6QXX9	BN5_0412	Polyhydroxyalkanoate synthase, class II PhaC2	CNA		CNA
W6QSQ8	BN5_0414	Poly(3-hydroxyalkanoate) polymerase PhaC1	4.04		3.73
W6QRB2	BN5_0438	Fis family transcriptional regulator	CNA		CNA
W6QQC6	BN5_0543	Transglutaminase domain-containing protein	13.10	8.11	
W6RBC9	BN5_0552	Urease accessory protein UreG	CNA	R	
W6QQF7	BN5_0578	Urease subunit Alpha	CNA	R	
W6QQH3	BN5_0593	ABC-type branched-chain amino acid transport system periplasmic component-like protein	15.71	8.70	
W6QQW2	BN5_0701	MerR family transcriptional regulator		R	R
W6QYV6	BN5_0702	Heavy metal translocating P-type ATPase		453.86	–130.94
W6QTN1	BN5_0704	Heavy metal transport/detoxification protein		R	R
W6RBV5	BN5_0715	Oligopeptide/dipeptide ABC transporter, ATPase	CNA		CNA
W6QZ51	BN5_0842	Beta-alanine-pyruvate transaminase		2.01	
W6QUE9	BN5_1006	Putative amino-acid ABC transporter-binding protein	6.92	4.24	
W6QZK3	BN5_1009	Putative ATP-binding component of a transporter	CNA		CNA
W6QUU3	BN5_2114	Nitrate transporter periplasmic component	CNA	R	–12.13
W6RFM9	BN5_2123	Assimilatory nitrite reductase	CNA	R	–32.97
W6R2U2	BN5_2125	Nitrate reductase	CNA	R	–24.11
W6QVA5	BN5_2229	Aminotransferase	12.42		6.18
W6QVA8	BN5_2234	Amidotransferase	CNA		CNA
W6QVB9	BN5_2244	Transglutaminase	CNA	R	2.03
W6QWV0	BN5_2413	GntR family transcriptional regulator	CNA	R	
W6QYJ4	BN5_2987	Branched-chain amino acid transport system substrate-binding protein	2.31		
W6QYM4	BN5_3018	Serine hydroxymethyltransferase	CNA		
W6QY54	BN5_3194	Spermidine/putrescine import ATP-binding protein	2.98		2.34
W6R622	BN5_3235	Periplasmic oligopeptide-binding protein	CNA	R	
W6QZC3	BN5_3236	Acetamidase/formamidase (EC 3.5.1.4)	CNA	R	
W6QZR6	BN5_3762	Polyamine-transporting ATPase (EC 3.6.3.31)	CNA		CNA
W6R077	BN5_3958	Glutamine synthetase	8.86	5.36	
W6RKV8	BN5_3962	Nitrogen regulation protein NR(I)	7.13	5.51	
W6R822	BN5_4009	Polyamine-transporting ATPase (EC 3.6.3.31)	CNA	R	2.54
W6R1F1	BN5_4010	Spermidine/putrescine-binding periplasmic protein 1	29.27	11.32	2.59

a Proteins marked with “CNA” or “R” indicate that they were exclusively identified in cells grown with 3-CNA or the jewelry residue, respectively.

The cysteine synthase homologs of *P. pseudoalcaligenes* CECT5344 were not upregulated by 3-CNA, while cells grown with the residue overproduced the cysteine synthase CysM3 (Table 1). Among proteins significantly upregulated by 3-CNA were a Fis-family transcriptional regulator, a serine hydroxymethyl transferase, the class II polyhydroxyalkanoate synthases PhaC1 and PhaC2, an aminotransferase and an amidotransferase, the glutamate synthase, and some polyamine and nitrogen compound transporters (Table 1). Proteins downregulated by 3-CNA were several subunits of the *cbb_3_*-type cytochrome *c* oxidase and the ATP synthase (Table S1).

Compared to ammonium, proteins upregulated by both 3-CNA and the cyanide-containing residue were, among others, a flavin monoamine oxidase, an acetamidase/formamidase, and proteins involved in transport and metabolism of alternative nitrogen sources (nitrate, urea, polyamines, and amino acids), such as assimilatory nitrate and nitrite reductases, urease, *N*-carbamoyl-l-amino acid amidohydrolase, glutamine synthetase, and transglutaminases, as well as the nitrogen regulatory protein NRI and a GntR family transcriptional regulator (Table 1). In contrast, glutamate dehydrogenase was downregulated by both 3-CNA and residue (Tables S1 and S2).

**FIG 2:**
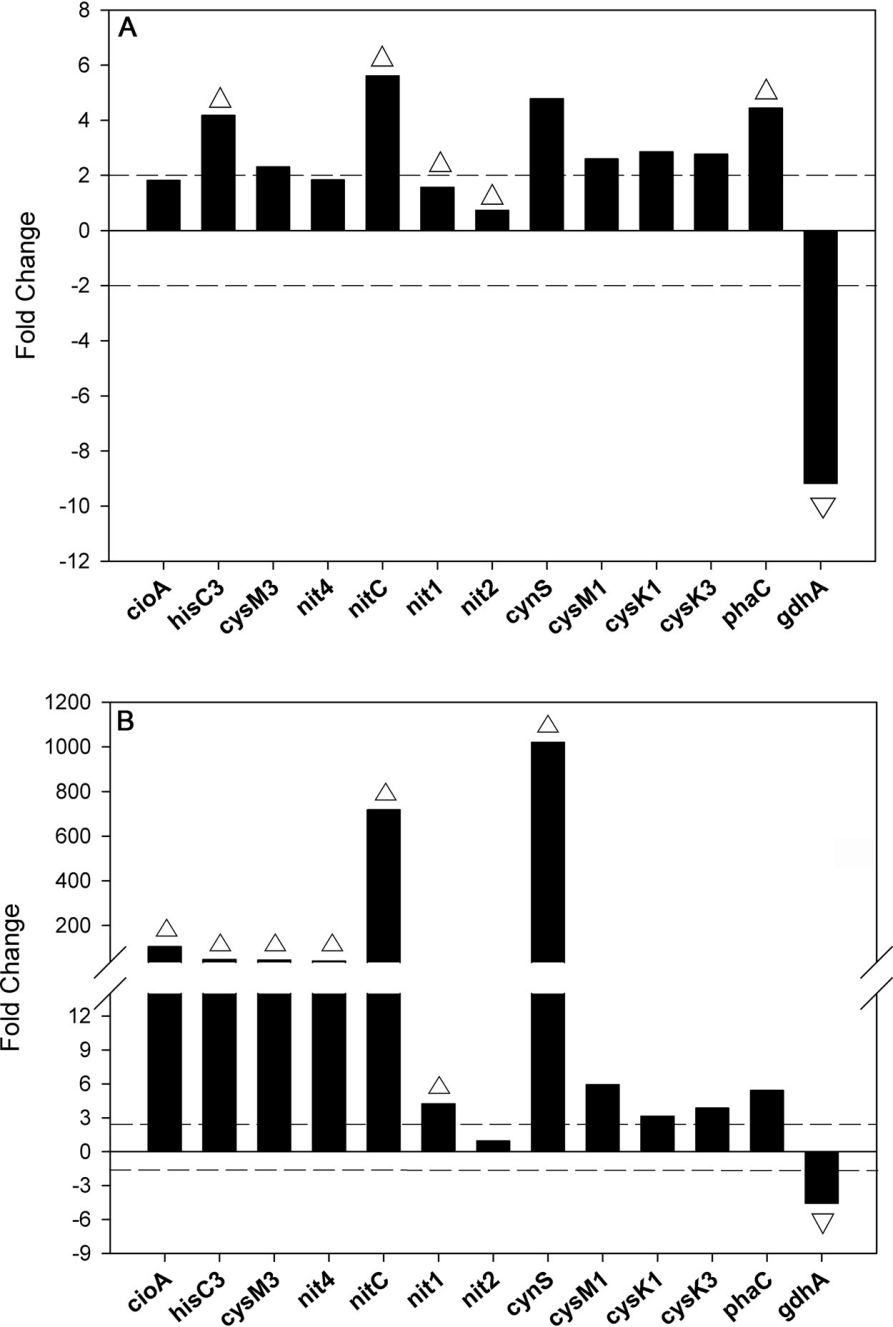
(A and B) Transcriptional analysis of *P. pseudoalcaligenes* CECT5344 genes encoding proteins differentially expressed in response to 3-CNA (A) or the cyanide-containing residue (B) with respect to ammonium. Cells were cultured with ammonium, 3-CNA, or the residue as the sole nitrogen source. The relative expression of different genes was determined using the *rpoB* gene for housekeeping. Data were obtained from 3 independent replicates, and fold changes were determined using ammonium as a reference. Only fold changes of ≥2 or ≤–2 were considered significant. Up- and down-pointing triangles indicate that the corresponding proteins were upregulated or downregulated, respectively, in the proteomic analysis (see Table 1).

To validate the proteomic data at the transcriptional level, a reverse-transcription quantitative PCR (qRT-PCR) analysis of 13 relevant genes, including some involved in cyanide, cyanate, or 3-CNA metabolism, was performed using RNA from *P. pseudoalcaligenes* CECT5344 cells grown with 3-CNA, jewelry residue, or ammonium (Fig. 2). The nitrilase *nitC* gene was significantly induced by both 3-CNA and the residue, compared to ammonium, while the *nit4* gene was only induced by the cyanide-containing wastewater. The *cioA*, *hisC3*, and *cysM3* genes, which are clustered with *nit4*, were also highly induced by the residue, while 3-CNA only induced *hisC3* gene expression. The *nit1* gene was slightly induced by the residue, but the *nit2* gene showed a basal expression. However, transcription of these two nitrilase genes was not significantly affected by 3-CNA. Expression of the cyanase *cynS* gene was induced at a much higher level in the cells grown with the residue than those grown with 3-CNA. This result was confirmed when cyanase activity was assayed in cell extracts from cells grown with the residue (628 U/mg) or 3-CNA (11 U/mg) as the sole N source. The genes *cysM1*, *cysK1*, and *cysK3* encoding putative cysteine synthases showed a slight induction in both the residue and 3-CNA with respect to ammonium. Finally, the polyhydroxyalkanoate synthase *phaC* gene was induced by both 3-CNA and the residue, whereas the glutamate dehydrogenase *gdhA* gene was repressed in both N sources (Fig. 2). In general, gene expression data correlated well with the proteomic results (Table 1, Fig. 2).

### The nitrilase NitC, but not the nitrilase Nit4, is required for 3-CNA assimilation under noncyanotrophic conditions.

**FIG 3:**
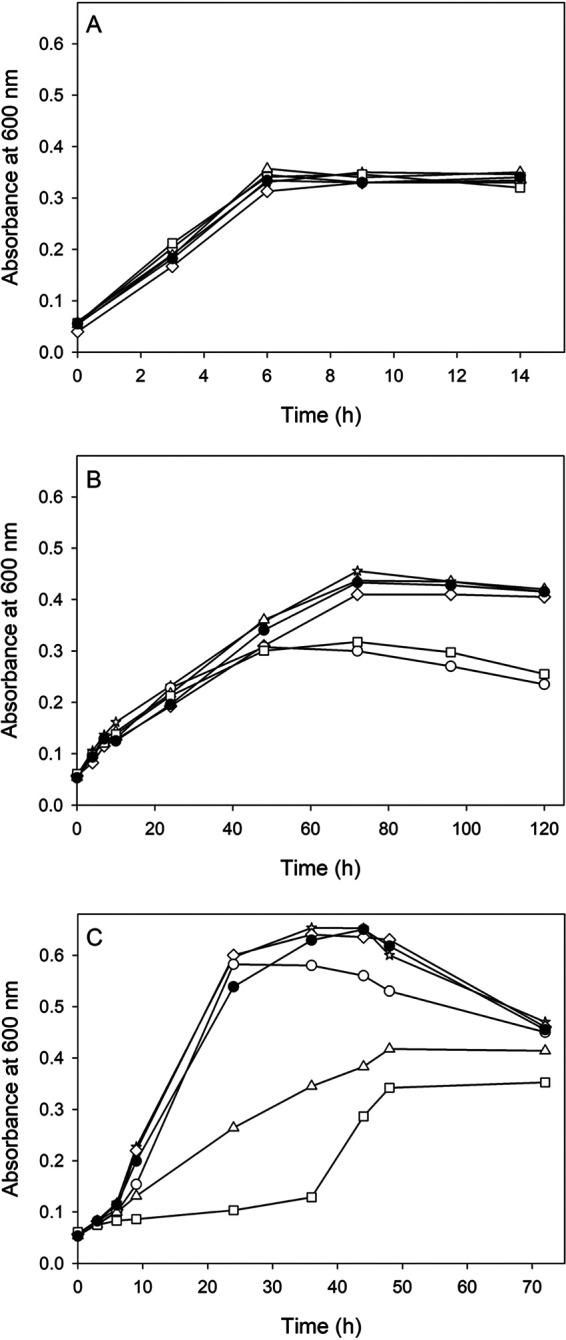
(A to C) Growth of *P. pseudoalcaligenes* CECT5344 wild-type and nitrilase-defective mutants in media with 2 mM ammonium (A), 2 mM 3-CNA (B), or 2 mM 3-CNA plus 1 mM NaCN (C) as the nitrogen source. Wild-type strain (filled circles), Nit1^−^ (stars), Nit2^−^ (diamonds), NitC^−^ (open circles), Nit4^−^ (triangles), and NitC^−^/Nit4^−^ (squares).

To characterize 3-CNA assimilation in the presence or absence of cyanide, four *P. pseudoalcaligenes* CECT5344 mutant strains defective in each nitrilase (NitC^−^, Nit4^−^, Nit1^−^, and Nit2^−^) and a double mutant, NitC^−^/Nit4^−^, were constructed and cultured with ammonium, 3-CNA, or 3-CNA plus cyanide. When ammonium was used as the N source, all strains showed similar growth rates (Fig. 3A). With 3-CNA as the sole N source, only the NitC^−^ and NitC^−^/Nit4^−^ mutants showed a phenotype different from the wild-type strain (Fig. 3B). At the initial stage, the growth rate was similar for all strains, but after 50 h of culture time, the growth of the NitC^−^ and NitC^−^/Nit4^−^ mutants stopped, reaching a maximal growth that was significantly lower than that in the rest of strains (Fig. 3B). However, all strains consumed 3-CNA at similar rates (not shown).

In medium with 3-CNA plus cyanide, the wild-type strain displayed a faster and higher growth than with 3-CNA alone (Fig. 3C). Under these cyanotrophic conditions, the NitC^−^, Nit1^−^, and Nit2^−^ mutants showed similar growth rates to the wild-type strain. It is important to highlight that the NitC^−^ mutant is unable to assimilate cyanide (7). Therefore, the increased growth of this mutant in medium containing 3-CNA plus NaCN with respect to 3-CNA (Fig. 3B and C) was only due to 3-CNA assimilation through the nitrilase Nit4, which was induced by cyanide. Nevertheless, in the presence of both 3-CNA and cyanide, the wild-type strain and the Nit1^−^ and Nit2^−^ mutants assimilate 3-CNA through Nit4, but they also assimilated cyanide through NitC, and this probably explains the slightly higher growth of these strains compared to the NitC^−^ mutant (Fig. 3C). Under these conditions, the Nit4^−^ mutant showed a lower growth rate than the wild-type strain, whereas growth of the NitC^−^/Nit4^−^ mutant had an extended lag phase of about 35 h (Fig. 3C). In addition, this double mutant reached about half of the maximal growth achieved by the rest of the strains under cyanotrophic conditions (3-CNA + CN^–^) but nearly the same maximal growth as with 3-CNA alone or with ammonium (Fig. 3).

**FIG 4:**
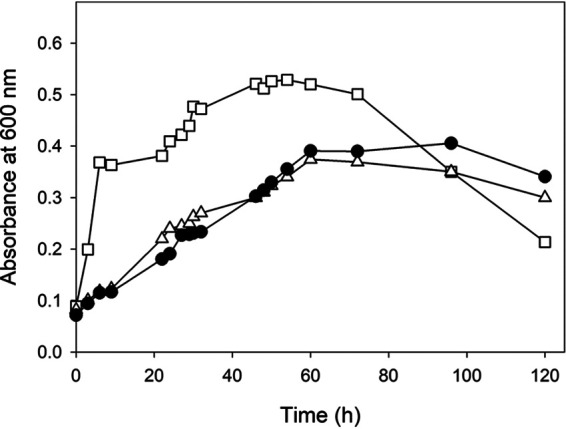
Growth of *P. pseudoalcaligenes* CECT5344 wild-type strain and MocR^−^ and MocR^−^/Nit4^−^ mutants with 2 mM 3-CNA as the sole nitrogen source. Wild-type strain (circles), MocR^−^ (squares), and MocR^−^/Nit4^−^ (triangles).

The *mocR* gene coding a putative GntR family transcriptional regulator is located directly upstream from, and with opposite orientation to, the *cio* gene cluster of *P. pseudoalcaligenes* CECT5344 (21). The quantitative proteomic analysis performed with a MocR^−^ mutant showed that proteins encoded by the *cio* gene cluster, including the nitrilase Nit4, were constitutively expressed, suggesting a negative regulatory role of MocR (unpublished data). In medium with 3-CNA as the sole N-source, this MocR^−^ mutant showed a both higher growth rate and a shorter lag phase than the wild-type strain (Fig. 4), probably due to the constitutive production of Nit4 even in the absence of cyanide. To test this hypothesis, a double mutant, MocR^−^/Nit4^−^, was constructed, and as expected, it showed the same phenotype as the wild-type strain in medium with 3-CNA (Fig. 4). On the other hand, a *P. pseudoalcaligenes* CECT5344 mutant in the dihydrodipicolinate synthase *dapA* gene, which is located upstream of *nit4* in the *cio* gene cluster, also constitutively expresses the nitrilase Nit4 as consequence of the antibiotic resistance cassette used in the construction of this mutant (30). This DapA^−^ mutant showed the same phenotype with 3-CNA as the sole N source as the MocR^−^ mutant (not shown).

## DISCUSSION

*P. pseudoalcaligenes* CECT5344 is an alkaliphilic bacterium that assimilates cyanide through the nitrilase NitC (6, 7). This strain also assimilates cyanate and 3-CNA (31, 32), but a CynS^−^ cyanase-defective mutant conserved the ability to assimilate cyanide, thus ruling out the involvement of cyanate in the cyanide assimilation pathway (28, 29). In addition, the failure to detect a 3-CNA synthase activity in *P. pseudoalcaligenes* CECT5344 cells grown with cyanide also indicates that 3-CNA could not be an intermediate in the cyanide assimilation pathway (22). However, the strain CECT5344 contains four putative cysteine synthase homologous genes, one of them (*cysM3*) clustered with the nitrilase *nit4* gene in the *cio* gene cluster required for cyanide resistance (21), thus suggesting the possibility of a vestigial cyanide degradation pathway through 3-CNA in this strain. Nevertheless, the CysM3 and CysM1 proteins of *P. pseudoalcaligenes* CECT5344 showed low identity with the P. putida 3-CNA synthase, revealing that these putative cysteine synthases may not function as 3-CNA synthases. The *cysK1* and *cysK3* genes, whose products displayed a high identity with the N- and C-terminal ends, respectively, of the P. putida 3-CNA synthase, showed the same gene expression profile, that is, a slight induction in both the residue and 3-CNA compared to ammonium (Fig. 2). These results, together with the sequence analysis (Fig. 1A), suggest that *cysK1* and *cysK3* could form part of the same gene, which lost its functionality as a consequence of the mutation that generated a premature stop codon. Although this gene could be expressed, its translation would generate a nonfunctional truncated protein corresponding to the first half of the gene (CysK1) that would be degraded by proteases, thus explaining the absence of the CysK1 and CysK3 proteins in the proteome.

The nitrilase Nit4 of *P. pseudoalcaligenes* CECT5344 may use 3-CNA as a substrate (23), but this enzyme is induced only by cyanide (24, 27). Therefore, exogenous 3-CNA could be assimilated through Nit4 in medium with cyanide, but the unsolved question is how 3-CNA is assimilated in the absence of cyanide, when Nit4 is not induced. In this work, a quantitative proteomic analysis by LC-MS/MS was carried out in *P. pseudoalcaligenes* CECT5344 cells grown with 3-CNA, a cyanide-containing jewelry residue, or ammonium as the sole N source (Table 1; Tables S1 to S3, Fig. S1 to S4). This proteomic analysis, the only one performed so far in response to 3-CNA, revealed that this nitrile has mainly an upregulatory effects on the proteome, affecting specifically, proteins involved in general nitrogen metabolism. This profile, and the upregulation of proteins involved in the biosynthesis of polyhydroxyalkanoates, suggests that 3-CNA is a poor nitrogen source in the absence of cyanide. In comparison to 3-CNA, the jewelry residue downregulated a high number of proteins due to the toxicity of metals and cyanide present in this wastewater. According to previous studies (24, 27), the jewelry residue upregulated proteins involved in cyanide and metal resistance mechanisms, including the cyanide-insensitive oxidase CioAB. In contrast, cells grown with 3-CNA did not upregulate CioAB or proteins involved in oxidative stress defense, highlighting that the molecular basis of cyanide and 3-CNA toxicity, and the resistance responses developed against these two cyano-compounds, are different in the strain CECT5344.

**FIG 5:**
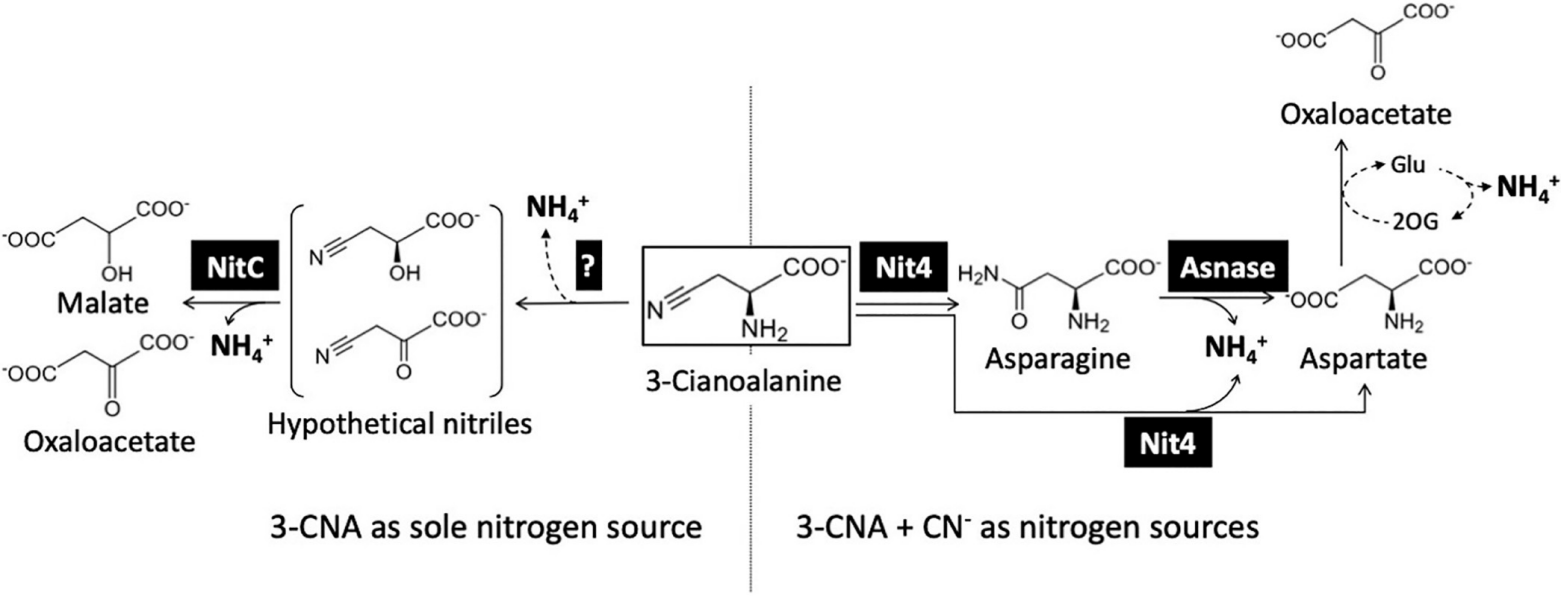
Proposed 3-CNA assimilation pathways in *P. pseudoalcaligenes* CECT5344 in the presence or absence of cyanide. In the absence of cyanide, *P. pseudoalcaligenes* CECT5344 assimilates 3-CNA by the NitC-dependent pathway (left) because the *nit4* gene is not induced under these conditions. When cyanide is present, the strain CECT5344 assimilates 3-CNA thought the Nit4-dependent pathway (right). Question mark, putative deaminase or aminotransferase; Asnase, asparaginase.

The *P. pseudoalcaligenes* CECT5344 genome contains four nitrilase genes, whose products belong to different phylogenetical branches (Fig. S5). The proteomic study revealed that nitrilases NitC, Nit1, and Nit2 were upregulated by 3-CNA. However, the nitrilase Nit4 was not induced by 3-CNA, although it was overexpressed in the cells grown with the cyanide-containing residue, like NitC and Nit1 (Table 1). This result was confirmed by a transcriptional qRT-PCR analysis (Fig. 2). This Nit4 expression profile is unusual because the 3-CNA nitrilase from Pseudomonas sp. 13 is induced by 3-CNA (18), and most bacterial nitrilases are induced by nitriles (33). Expression of the *P. pseudoalcaligenes* CECT5344 *nit4* gene could be related to its location in the *cio* gene cluster, which is specifically induced by cyanide and not by 3-CNA (Fig. 2). Curiously, bacterial *nit4* homologs with a similar gene context have not been described up to date. In previous studies, the essential role of the nitrilase NitC in the assimilation of cyanide was highlighted (7). Induction of NitC by 3-CNA suggests the possible involvement of this nitrilase in 3-CNA assimilation (Table 1 and Table S1). A mutational analysis revealed that the additional nitrilases Nit1 and Nit2 could not have an essential role in the assimilation of 3-CNA, with or without cyanide (Fig. 3). We propose that 3-CNA may be assimilated in *P. pseudoalcaligenes* CECT5344 through two different metabolic pathways, using either Nit4 or NitC, depending on cyanide availability (Fig. 5). Nit4 is relevant for 3-CNA assimilation when cyanide is present (cyanotrophic conditions), while NitC is required to assimilate 3-CNA in the absence of cyanide (noncyanotrophic conditions).

It has been described that Nit4 has dual nitrilase/nitrile hydratase activity and uses 3-CNA as a substrate, forming either aspartate plus ammonium or asparagine, which may be further hydrolyzed to aspartate and ammonium by an asparaginase (23). Thus, the wild-type strain CECT5344 may obtain by this route up to two ammonium molecules for each 3-CNA molecule (Fig. 5, right). However, proteomic and transcriptomic analyses suggest that this 3-CNA assimilation route is not functional in the absence of cyanide, because the *nit4* gene is induced by the cyanide-containing residue, but not by 3-CNA as it occurs in other organisms. Under noncyanotrophic conditions, 3-CNA is assimilated through the nitrilase NitC, although this enzyme could not directly use 3-CNA as a substrate, but rather, used the nitrile formed after 3-CNA deamination/transamination (Fig. 5, left). By this alternative pathway, which may be active regardless of the presence of cyanide since NitC is overexpressed with both 3-CNA and the cyanide-containing wastewater (Table 1, Fig. 2), the wild-type strain may also obtain two ammonium molecules for each 3-CNA molecule. The limiting factor of this alternative route would be the 3-CNA deamination/transamination step that generates the nitrile acting as the substrate for NitC. Among the proteins overexpressed with 3-CNA (Table 1), different aminotransferases and a flavin monoamine oxidase could be putative candidates for catalyzing the first reaction of this novel pathway for 3-CNA assimilation, although other enzymes expressed constitutively cannot be discarded.

The phenotypes of all nitrilase-defective single mutants (Fig. 3) may be adequately explained by the alternative metabolic pathways proposed in Fig. 5. In the NitC^−^/Nit4^−^ double mutant, the Nit4 pathway is never functional, but the alternative NitC pathway may allow bacterial growth, although obtaining only one ammonium molecule in the first deamination/transamination step. This may explain that in the absence of cyanide, a condition in which the nitrilase Nit4 is not expressed, the NitC^−^/Nit4^−^ and NitC^−^ mutants showed similar growth, which was lower than in the wild-type strain. Nonetheless, when cyanide was present, the growth of the double mutant was lower and slower than in the wild-type strain and the single NitC^−^ mutant (Fig. 3). In addition, the growth curves with 3-CNA of the MocR^−^ and MocR^−^/Nit4^−^ mutants (Fig. 4) also support the existence of two routes for 3-CNA assimilation in *P. pseudoalcaligenes* CECT5344 (Fig. 5). The growth of the wild-type strain with 3-CNA as the sole nitrogen source was due to the pathway comprising the putative deaminase/aminotransferase and the nitrilase NitC (Fig. 5, left). The absence of the putative repressor MocR, and the consequent overexpression of the nitrilase Nit4, allowed a higher and faster growth of the MocR^−^ mutant strain through the Nit4 pathway (Fig. 5, right). This was also supported by the MocR^−^/Nit4^−^ double mutant, which showed the same phenotype as the wild-type strain due to the absence of Nit4 (Fig. 4). Furthermore, similar results have been observed in a *P. pseudoalcaligenes* DapA^−^ mutant that constitutively expresses the nitrilase *nit4* gene from the promoter of the antibiotic resistance cassette inserted in this mutant (30).

In summary, assimilation of 3-CNA in *P. pseudoalcaligenes* CECT5344 differs depending on the presence or absence of cyanide. Under cyanotrophic conditions, with both cyanide and 3-CNA, cyanide induces expression of the *nit4* gene, and 3-CNA is assimilated via asparagine/aspartate by the nitrilase Nit4 (Fig. 5, right). Additionally, cyanide may be also assimilated by NitC. In the absence of cyanide, when the *nit4* gene is not induced, the strain CECT5344 assimilates 3-CNA through the NitC pathway without formation of asparagine and aspartate as intermediates (Fig. 5, left). This alternative route constitutes a more general pathway for 3-CNA assimilation because it may be functional both in the presence and in the absence of cyanide. These two 3-CNA assimilation pathways in *P. pseudoalcaligenes* CECT5344 could have their origin in an ancestral cyanide degradation pathway through 3-CNA as the intermediate that lost its functionality as a consequence of a point mutation in the archaic *cysK* gene encoding a 3-CNA synthase. The possible coexistence in the past of two cyanide-degradation pathways for cyanide assimilation in the strain CECT5344, through the nitrilases NitC and Nit4, suggests that cyanide could act as an important selective pressure in the original niche of this cyanotrophic bacterium.

## MATERIALS AND METHODS

### Chemicals.

All chemicals used in this study were analytical grade and were purchased from Sigma-Aldrich (St. Louis, MO, USA). The jewelry industry wastewater, provided by Gemasur S.L. (Córdoba, Spain), contains 1.5 M cyanide and copper, iron, and zinc as the most representative metals (27). Wastes containing cyanide were disposed of by the Environmental Protection Unit, University of Córdoba (UCO).

### Bacterial strains, plasmids, and growth conditions.

Wild-type and mutant strains of *P. pseudoalcaligenes* CECT5344 (Table 2) were grown in Erlenmeyer flasks filled to 20% of their total volume with M9 minimal medium (34), adjusted to pH 9.5, under aerobic conditions at 30°C and 220 rpm (22). Sodium acetate (50 mM) was used as the carbon source, and the different nitrogen sources, ammonium chloride, sodium cyanide, 3-CNA, and jewelry wastewater, were added to the medium at the indicated concentrations. Cells were harvested by centrifugation at 4,500 × *g* for 10 min when about 40 to 50% of the nitrogen source was consumed (35), and the pellets were kept at −80°C until use.

**TABLE 2:** Bacterial strains used in this study^*a*^

Strain	Characteristics	Reference
*P. pseudoalcaligenes* strains		
CECT5344	Wild type; uses cyanide as N source	22
DapA1^—^	Gm-directed mutant in the *dapA1* gene	30
NitC^—^	Gm-directed mutant in the *nitC* gene	7
Nit1^—^	Gm-directed mutant in the *nit1* gene	This work
Nit2^—^	Km-directed mutant in the *nit2* gene	This work
Nit4^—^	Km-directed mutant in the *nit4* gene	This work
MocR^—^	Gm-directed mutant in the *mocR* gene	This work
NitC^—^/ Nit4^—^	Gm- and Km-directed double mutant in the *nitC* and *nit4* genes, respectively	This work
MocR^—^/ Nit4^—^	Gm- and Km-directed double mutant in the *mocR* and *nit4* genes, respectively	This work
E. coli strains		
DH5α	Lac^–^; host for most plasmids	36
S17-1	Tra^+^; host for the mobilizable *mob* plasmids	45

a Gm, gentamicin; Km, kanamycin.

Escherichia coli strains (Table 2) were grown in Luria-Bertani (LB) medium (36) at 37°C and 220 rpm on a rotatory shaker. When solid agar plates were required, 1.5% bacteriological agar was added. Ampicillin (100 mg/ml), kanamycin (25 mg/ml), nalidixic acid (10 mg/ml), or gentamicin (20 mg/ml) was supplied depending on the requirements of the strains (35).

### Analytical determination and enzyme activity assays.

Bacterial growth was monitored by following the absorbance of the cultures at 600 nm. Ammonium, free cyanide, and protein concentration were determined by standard methods (37–39). Cyanase activity was assayed as previously described (29). 3-CNA was quantified by high-pressure liquid chromatography (HPLC) analysis using a Thermo Scientific Hypersil octyldecyl silane (ODS) C_18_ column (5 μm diameter particle size, 4.6 × 250 mm). Mobile phases were solvent A (0.5% acetic acid and 0.0075% triethylamine in deionized water), which was degassed under reduced pressure and filtered with a 0.45-μm filter (Millipore, USA), and solvent B (100% acetonitrile). The gradient was 0 to 10 min, 17.5% B; 10 to 40 min, 35% B; and 40 to 45 min, 40% B. Chromatographic conditions were flow rate, 1 ml/min; column temperature, 35°C; detection, 254 nm; and injection, 20 μl. To prepare a calibration curve, a 3-CNA 0.5 M solution (in water with 2 N HCl) was diluted to 25, 50, 75, and 100 μM concentrations. Dansylation was carried out as previously described (40). Aliquots from the cultures with 3-CNA were centrifuged (4,500 × *g*, 10 min), and the supernatants were mixed with 0.25% ethanol, 1% (w/v) NaHCO_3_ in deionized water, and 10% (w/v) dansyl chloride in acetonitrile. Samples were kept at −80°C until use.

### Generation of mutant strains of *P. pseudoalcaligenes* CECT5344.

Mutants were constructed by insertion of an antibiotic resistance cassette into a central region of the corresponding gene. Total genomic DNA was isolated from *P. pseudoalcaligenes* CECT5344 using a commercial kit (Canvax, Córdoba, Spain). For plasmid DNA isolation, a FavorPrep plasmid mini-extraction kit (FAPDE300; Favorgen) was used according to the protocol supplied. Restriction enzyme digestions were carried out using the New England Biolabs reagents, and DNA fragments were run on 1% agarose gel in TAE buffer (40 mM Tris–HCl, 20 mM acetic acid, and 1 mM EDTA). Ligations and DNA manipulations were performed according to standard methods (34). Sequencing of DNA fragments was performed at the Genomic Unit at the Central Service for Research Support (SCAI, UCO). The *P. pseudoalcaligenes* CECT5344 genome sequence is available on the EMBL database under the accession no. LK391695.

To generate the Nit1^−^ mutant, the BN5_1925 gene was amplified from genomic DNA by PCR using specific oligonucleotides (Table S4), and the obtained 1,431-bp DNA fragment was cloned into pGEM-T Easy. A gentamicin resistance cassette was inserted into the SalI site of this fragment. The *nit1*::*Gm* fragment was subcloned into the pK18*mob* suicide vector using EcoRI and BamHI sites, and the final pK18*mob*-*nit1*::*Gm* plasmid was transferred by conjugation from E. coli S17-1 (donor strain) to *P. pseudoalcaligenes* CECT5344 (nalidixic acid-resistant receptor strain). Transconjugants were selected with nalidixic acid and gentamicin.

To construct a Nit2^−^ mutant, the BN5_4427 gene was disrupted by insertion of the kanamycin-resistant pK18*mob*. A 415-bp DNA fragment was amplified by PCR with the indicated oligonucleotides (Table S4) and cloned into pGEM-T Easy and later into pK18*mob* using BamHI and EcoRI restriction sites. The pK18*mob*-*nit2* plasmid was transferred by conjugational mating from E. coli S17-1 to *P. pseudoalcaligenes* CECT5344, and transconjugants were selected with nalidixic acid and kanamycin.

The Nit4^−^ mutant was generated by insertion of a kanamycin cassette into the BN5_1912 gene, which was amplified by PCR using specific oligonucleotides (Table S4). The resulting 512-bp DNA fragment was cloned into pGEM-T Easy and subcloned into pK18*mob* as a EcoRI-BamHI fragment. The pK18*mob*::*nit4* plasmid was transferred by conjugation from E. coli S17-1 to *P. pseudoalcaligenes* CECT5344, and the mutant was selected with nalidixic acid and kanamycin.

The MocR^−^ mutant was constructed by partial deletion of the *gntR/mocR* gene (BN5_1899) and insertion of the gentamicin resistance cassette. Two regions of this gene, separated by 592 bp, were amplified by PCR with the indicated oligonucleotide pairs (Table S4). The obtained 432-bp and 537-bp fragments were cloned into pGEM-T Easy. A gentamicin resistance cassette was inserted into the BamHI restriction site generated between both fragments. This construction was subcloned into pK18*mob*, and the pK18*mob-*mocR::*Gm* plasmid was transferred from E. coli S17-1 to *P. pseudoalcaligenes* CECT5344 by mating. The mutant was selected with nalidixic acid and gentamicin.

The NitC^−^ and DapA^−^ mutants were previously reported (7, 30). Generation of the NitC^−^/Nit4^−^ and MocR^−^/Nit4^−^ double mutants was performed as described for Nit4^−^ but using the NitC^−^ or MocR^−^ mutant, respectively, as receptor strain, and selecting the transconjugants with gentamicin and kanamycin.

The authenticity of all mutants was confirmed by PCR analysis and DNA sequencing. The kanamycin and gentamicin resistance cassettes used in this work, which do not contain any transcriptional stop, were inserted with the same orientation as the target genes, discarding a possible polar effect over the downstream genes in the different mutants constructed, as previously described (7).

### Proteomic analysis by liquid chromatography coupled to mass spectrometry (LC-MS/MS).

*P*. *pseudoalcaligenes* CECT5344 cells were grown in M9 minimal medium containing 50 mM sodium acetate as the carbon source and 2 mM ammonium chloride, 2 mM 3-CNA, or jewelry residue (2 mM free cyanide) as the N source. When about 50% of the total nitrogen was consumed (about at the middle of the log phase), cells were harvested by centrifugation at 4,500 × *g* for 10 min at 4°C, and pellets were kept at −80°C until use. Three biological replicates were cultured for each nitrogen source to perform the quantitative proteomic LC-MS/MS analysis (Proteomic Unit, SCAI, UCO), as previously described (30, 41). Proteomic data were deposited in the ProteomeXchange Consortium (http://proteomecentral.proteomexchange.org) via the PRIDE partner repository (42) with the data set identifier PXD026640.

### RNA quantitation by qRT-PCR.

RNA isolation, cDNA synthesis, and quantitation were carried out with wild-type cells grown with NH_4_Cl, 3-CNA, or cyanide-containing jewelry residue as the sole nitrogen source, as previously described (30). Target cDNAs were amplified in three independent PCRs using specific primer pairs (Table S4). For relative quantitation of the fluorescence values, a calibration curve was performed using dilution series from 80 to 0.008 ng of *P. pseudoalcaligenes* CECT5344 genomic DNA. Data were normalized by using the housekeeping *rpoB* gene.

### Bioinformatic analysis and statistics.

Growth curves and analytical determinations were carried out three times in separate experiments, with mean standard deviations never exceeding 5%. Oligo 7 software was used for primer design. Data from the transcriptional qRT-PCR analysis were subjected to a *t* test. The proteomic analysis was carried out using MaxQuant (43), and data were analyzed using the free software Perseus 1.5.6.02. Proteins identified from only one peptide were discarded, and for differential pairwise comparisons, only proteins identified in at least two replicates under each condition were considered. Then, statistical significance was analyzed using a two-tailed *t* test, and proteins were considered differentially expressed when the absolute value of the fold change was ≥2 with a *P* value of <0.05. Proteins identified only in one N source were considered exclusive from that condition (30, 41). GO analysis was performed using Comparative GO web (25). Only changes with a *P* value of <0.05 after a hypergeometric distribution [E(GO)] test of the third level of GO biological function were shown. The whole genome of *P. pseudoalcaligenes* CECT5344 was used as a reference, and the parameter [E(GOi)], which indicates the GO enrichment, was calculated with the formula [E(GOi)] = sample size/genome size × GOi. Integration of final proteomic data was performed using the tool KEGG Mapper (44). For the phylogenetical analysis, MUSCLE and PhyML were used.
