# Supplementary material for: Alternative Pathway for 3-Cyanoalanine Assimilation in Pseudomonas pseudoalcaligenes CECT5344 under Noncyanotrophic Conditions
Source: Microbiol Spectr. 2021 Nov 3;9(3):e00777-21. doi: 10.1128/Spectrum.00777-21 (PMC8567248; doi:10.1128/Spectrum.00777-21)
Supplement: SUPPLEMENTAL FILE 1 — Supplemental material. Download SPECTRUM00777-21_Supp_1_seq11.pdf, PDF file, 1.2 MB [file spectrum00777-21_supp_1_seq11.pdf]

## SUPPLEMENTAL MATERIAL

**Table S1**. Proteins differentially expressed in *P. pseudoalcaligenes* CECT5344 in response to 3-CNA using ammonium as control. Proteins significantly up-regulated (fold change ≥ 2) and down-regulated (fold change ≤ 2) are shown. "CNA" or "N" indicates that proteins were detected exclusively in cells grown with 3-CNA or ammonium, respectively.

| Uniprot ID | Gene locus | Protein Name | Fold Change 3-CNA/N | *p* value |
|---|---|---|---|---|
| W6R1F1 | BN5_4010 | Spermidine/putrescine-binding periplasmic protein 1 | 29.27 | 0.00902 |
| W6QXC5 | BN5_0151 | Flavin monoamine oxidase-related protein (EC 1.4.3.4) | 18.90 | 0.001126 |
| W6QQH3 | BN5_0593 | ABC-type branched-chain amino acid transport systems periplasmic component-like protein | 15.71 | 9.68E-05 |
| W6QQC6 | BN5_0543 | Transglutaminase domain-containing protein | 13.10 | 0.000153 |
| W6QVA5 | BN5_2229 | Aminotransferase (EC 2.6.1.-) | 12.42 | 0.005628 |
| W6RKJ4 | BN5_3761 | Extracellular solute-binding protein | 11.33 | 4.92E-05 |
| W6RAC3 | BN5_0149 | Putative endoribonuclease L-PSP | 10.42 | 0.003533 |
| W6QWF0 | BN5_2246 | Uncharacterized protein | 9.06 | 0.00036 |
| W6RC44 | BN5_0830 | Basic membrane lipoprotein | 8.92 | 0.004173 |
| W6R077 | BN5_3958 | Glutamine synthetase (EC 6.3.1.2) | 8.86 | 0.000307 |
| W6R374 | BN5_2245 | Uncharacterized protein | 8.28 | 0.013879 |
| W6QPA6 | BN5_0150 | Cytochrome C6 | 7.50 | 0.00221 |
| W6RKV8 | BN5_3962 | Nitrogen regulation protein NR(I) | 7.13 | 0.010376 |
| W6QUE9 | BN5_1006 | Putative amino-acid ABC transporter-binding protein | 6.92 | 4.76E-05 |
| W6QTP6 | BN5_0719 | Extracellular solute-binding protein | 6.89 | 0.000352 |
| W6R0L4 | BN5_3207 | Basic membrane protein A2 Immunodominant antigen P39 | 6.70 | 0.013968 |
| W6QRZ0 | BN5_1113 | Glycerol-3-phosphate dehydrogenase (EC 1.1.5.3) | 6.15 | 0.000353 |
| W6R989 | BN5_4427 | Bifunctional nitrilase/nitrile hydratase NIT4B (EC 3.5.5.1) | 5.30 | 0.000125 |
| W6RL49 | BN5_4062 | Uncharacterized protein | 4.19 | 3.32E-05 |
| W6QSQ8 | BN5_0414 | Poly(3-hydroxyalkanoate) polymerase (EC 2.3.1.-) | 4.04 | 0.002035 |
| W6QYV0 | BN5_3096 | Porin D (EC 3.4.21.-) | 3.99 | 0.000201 |
| W6RLB1 | BN5_4130 | Putative tyrosine-protein kinase epsB (EC 2.7.10.-) | 3.57 | 0.002342 |
| W6QSB1 | BN5_0783 | Alcohol dehydrogenase (EC 1.-.-.-) | 3.44 | 0.00214 |
| W6QUT3 | BN5_1646 | Small heat shock protein | 3.41 | 4.96E-06 |
| W6R5D0 | BN5_3027 | ABC-type Fe3+ transport system periplasmic component-like protein | 3.03 | 0.010231 |
| W6QY54 | BN5_3194 | Spermidine/putrescine import ATP-binding protein potA (EC 3.6.3.31) | 2.98 | 0.001015 |
| W6QTK8 | BN5_1649 | ATP-dependent zinc metalloprotease FtsH (EC 3.4.24.-) | 2.89 | 0.000135 |
| W6RAR6 | BN5_0330 | Dihydropyrimidinase (EC 3.5.2.2) | 2.69 | 0.002578 |
| W6R0J6 | BN5_4079 | Membrane-bound lytic murein transglycosylase F | 2.65 | 0.002607 |
| W6R1J1 | BN5_1650 | Diguanylate cyclase/phosphodiesterase with PAS/PAC and GAF sensor(S) | 2.60 | 0.011154 |
| W6RE08 | BN5_1524 | Fumarate hydratase class II (Fumarase C) (EC 4.2.1.2) | 2.51 | 0.00211 |
| W6QZT0 | BN5_2953 | Outer membrane porin F | 2.47 | 0.000784 |
| W6R104 | BN5_1497 | Threonine aldolase, low-specificity (EC 4.1.2.5) | 2.42 | 0.002792 |
| W6R2P1 | BN5_2079 | Uncharacterized protein | 2.41 | 0.032577 |
| W6QP71 | BN5_0124 | Malate synthase G (EC 2.3.3.9) | 2.40 | 0.001647 |
| W6QXK5 | BN5_0267 | Polyamine-transporting ATPase (EC 3.6.3.31) | 2.38 | 0.00169 |
| W6RJX5 | BN5_3557 | SMC domain-containing protein | 2.38 | 0.02707 |

| | | | | |
|---|---|---|---|---|
| W6RDM2 | BN5_1373 | MOSC domain-containing protein 1 (EC 1.-.-.-) | 2.38 | 0.01127 |
| W6QW62 | BN5_1647 | ATP-dependent Clp protease ATP-binding subunit ClpC (EC 3.1.21.-) | 2.37 | 0.001478 |
| W6R782 | BN5_3669 | Catalase-peroxidase (CP) (EC 1.11.1.21) (Peroxidase/catalase) | 2.36 | 0.002267 |
| W6QY03 | BN5_0432 | Type IV pilus assembly protein PilQ | 2.35 | 0.000493 |
| W6RE45 | BN5_1567 | Bacterial luciferase family protein (EC 1.14.-.-) | 2.35 | 0.00041 |
| W6RKY3 | BN5_3997 | Aldehyde dehydrogenase | 2.35 | 0.001177 |
| W6RB40 | BN5_0465 | Pyruvate dehydrogenase E1 component (EC 1.2.4.1) | 2.31 | 0.001132 |
| W6QYJ4 | BN5_2987 | Branched-chain amino acid transport system substrate-binding protein | 2.31 | 0.003671 |
| W6R2G4 | BN5_3896 | NADPH:quinone oxidoreductase 1 (EC 1.6.5.2) | 2.28 | 0.012844 |
| W6QRD3 | BN5_0876 | Acetyl-CoA acetyltransferase (EC 2.3.1.9) | 2.27 | 0.008966 |
| W6QS11 | BN5_0127 | Glycolate oxidase FAD binding subunit (EC 1.1.3.15) | 2.25 | 0.004133 |
| W6R036 | BN5_1154 | Uncharacterized protein | 2.23 | 0.001496 |
| W6RJ11 | BN5_3198 | Cyanuric acid amidohydrolase (EC 3.5.2.15) | 2.21 | 1.72E-05 |
| W6QPL1 | BN5_0266 | Putrescine transport system substrate-binding protein | 2.21 | 0.000791 |
| W6R0Q0 | BN5_4131 | Putative capsule polysaccharide export protein | 2.20 | 0.002776 |
| W6QZS8 | BN5_1079 | Thiol:disulfide interchange protein DsbC (EC 5.3.4.1) | 2.19 | 0.029955 |
| W6QYW7 | BN5_3493 | GTP cyclohydrolase 1 type 2 homolog | 2.18 | 0.004652 |
| W6RAL1 | BN5_0265 | Putrescine transporter periplasmic protein | 2.18 | 0.010339 |
| W6RKK5 | BN5_3776 | Putative serine protein kinase, PrkA | 2.17 | 0.005105 |
| W6QY80 | BN5_2877 | Acyl-CoA synthetase (EC 6.2.1.3) | 2.11 | 0.020333 |
| W6R2R6 | BN5_4453 | Uncharacterized protein | 2.11 | 0.020883 |
| W6QXS8 | BN5_2222 | Peptide ABC transporter, periplasmic peptide-binding protein | 2.11 | 0.005626 |
| W6R0M8 | BN5_1380 | 3-ketoacyl-CoA thiolase (EC 2.3.1.16) (Acetyl-CoA acyltransferase) (Beta-ketothiolase) (Fatty acid oxidation complex subunit beta) | 2.10 | 0.006996 |
| W6R155 | BN5_3910 | Acyl-CoA dehydrogenase domain-containing protein (EC 1.3.99.-) | 2.08 | 0.004137 |
| W6QX86 | BN5_2542 | Aconitate hydratase (Aconitase) (EC 4.2.1.3) | 2.06 | 0.00482 |
| W6QZM8 | BN5_1034 | Zinc-containing alcohol dehydrogenase superfamily (EC 1.1.1.1) | 2.06 | 0.019585 |
| W6QSM0 | BN5_0374 | Substrate-binding region of ABC-type glycine betaine transport system | 2.02 | 0.004894 |
| W6QYH1 | BN5_2967 | dCTP deaminase (EC 3.5.4.13) (Deoxycytidine triphosphate deaminase) | 2.00 | 0.019621 |
| W6R9G5 | BN5_4497 | ATP synthase subunit beta (EC 3.6.3.14) (ATP synthase F1 sector subunit beta) (F-ATPase subunit beta) | -2.00 | 0.012385 |
| W6RKF5 | BN5_3697 | 30S ribosomal protein S10 | -2.02 | 0.014467 |
| W6QYL1 | BN5_3372 | 50S ribosomal protein L25 (General stress protein CTC) | -2.03 | 0.002583 |
| W6REP1 | BN5_1748 | Trigger factor (TF) (EC 5.2.1.8) (PPIase) | -2.03 | 0.013627 |
| W6QZG2 | BN5_2833 | Elongation factor Ts (EF-Ts) | -2.03 | 0.006244 |
| W6R1Y8 | BN5_3701 | 30S ribosomal protein S12 | -2.06 | 0.003883 |
| W6QQU7 | BN5_0278 | Resolvase | -2.06 | 0.001294 |
| W6QXP5 | BN5_0322 | ATP-dependent RNA helicase RhlE (EC 3.6.4.13) | -2.06 | 0.00047 |
| W6QQH7 | BN5_0598 | Phosphoribosylamine--glycine ligase (EC 6.3.4.13) (GARS) (Glycinamide ribonucleotide synthetase) (Phosphoribosylglycinamide synthetase) | -2.07 | 0.014492 |
| W6QPN0 | BN5_0286 | 33 kDa chaperonin (Heat shock protein 33 homolog) (HSP33) | -2.10 | 0.002295 |
| W6R2W1 | BN5_4498 | ATP synthase gamma chain (ATP synthase F1 sector gamma subunit) (F-ATPase gamma subunit) | -2.14 | 0.003105 |
| W6QZF3 | BN5_3673 | 30S ribosomal protein S11 | -2.17 | 0.023631 |
| W6R1W4 | BN5_3691 | 50S ribosomal protein L22 | -2.18 | 0.013758 |
| W6QP53 | BN5_0119 | Phosphate-specific transport system accessory protein PhoU | -2.18 | 0.012711 |
| W6R1S5 | BN5_4496 | ATP synthase epsilon chain (ATP synthase F1 sector epsilon subunit) (F-ATPase epsilon subunit) | -2.20 | 0.012936 |
| W6R168 | BN5_3925 | Malate synthase G (EC 2.3.3.9) | -2.21 | 0.001359 |

| | | | | |
|---|---|---|---|---|
| W6QVX3 | BN5_1543 | Ribosomal protein S12 methylthiotransferase RimO (S12 MTTase) (S12 methylthiotransferase) (EC 2.8.4.4) (Ribosomal protein S12 (aspartate-C(3))-methylthiotransferase) (Ribosome maturation factor RimO) | -2.21 | 0.029528 |
| W6RJL0 | BN5_3457 | Cell division protein FtsZ | -2.28 | 0.000106 |
| W6QSW2 | BN5_0985 | Saccharopine dehydrogenase (EC 1.5.1.7) | -2.30 | 0.005949 |
| W6R9H1 | BN5_4502 | ATP synthase subunit c | -2.34 | 0.003953 |
| W6R0I3 | BN5_3670 | 50S ribosomal protein L17 | -2.36 | 0.011325 |
| W6RKB4 | BN5_3682 | 30S ribosomal protein S8 | -2.37 | 0.001522 |
| W6R1X7 | BN5_4198 | Laminin subunit gamma-1 | -2.38 | 0.029022 |
| W6QVW1 | BN5_2446 | Cytochrome c oxidase, cbb3-type, subunit II (EC 1.9.3.1) | -2.39 | 0.009391 |
| W6R648 | BN5_3281 | Uncharacterized protein | -2.40 | 0.031909 |
| W6RJ83 | BN5_3304 | Uncharacterized protein | -2.41 | 0.014647 |
| W6R197 | BN5_3491 | Adenylyl-sulfate kinase (EC 2.7.1.25) (EC 2.7.7.4) (APS kinase) (ATP adenosine-5'-phosphosulfate 3'-phosphotransferase) (ATP-sulfurylase large subunit) (Adenosine-5'-phosphosulfate kinase) (Sulfate adenylate transferase) (SAT) (Sulfate adenylyltransferase subunit 1) | -2.44 | 0.005591 |
| W6REY3 | BN5_1853 | Isocitrate lyase (EC 4.1.3.1) | -2.44 | 0.015809 |
| W6R1C9 | BN5_4326 | D-methionine-binding lipoprotein metQ | -2.47 | 0.000654 |
| W6R0P7 | BN5_3705 | 50S ribosomal protein L10 | -2.47 | 0.024309 |
| W6RKD3 | BN5_3687 | 30S ribosomal protein S17 | -2.55 | 0.00991 |
| W6QVT3 | BN5_2025 | Uncharacterized protein | -2.58 | 0.004059 |
| W6R7C1 | BN5_3694 | 50S ribosomal protein L23 | -2.61 | 0.014851 |
| W6QWY4 | BN5_2448 | Cbb3-type cytochrome c oxidase subunit | -2.71 | 0.004478 |
| W6R0J3 | BN5_3680 | 50S ribosomal protein L18 | -2.73 | 0.010259 |
| W6R473 | BN5_4434 | Peptide methionine sulfoxide reductase MsrA (Protein-methionine-S-oxide reductase) (EC 1.8.4.11) (Peptide-methionine (S)-S-oxide reductase) (Peptide Met(O) reductase) | -2.78 | 0.000616 |
| W6QZL4 | BN5_3693 | 50S ribosomal protein L2 | -2.91 | 0.000297 |
| W6QZU8 | BN5_2968 | Cold shock protein (Beta-ribbon, CspA family) | -2.92 | 0.015161 |
| W6R1T2 | BN5_4501 | ATP synthase subunit b (ATP synthase F(0) sector subunit b) (ATPase subunit I) (F-type ATPase subunit b) (F-ATPase subunit b) | -3.00 | 0.033557 |
| W6QT34 | BN5_0536 | 30S ribosomal protein S18 | -3.05 | 0.016286 |
| W6RA44 | BN5_0118 | Phosphate import ATP-binding protein PstB (EC 3.6.3.27) (ABC phosphate transporter) (Phosphate-transporting ATPase) | -3.37 | 0.005045 |
| W6QWP5 | BN5_2353 | Uncharacterized protein | -3.39 | 0.002236 |
| W6QS66 | BN5_0209 | Alginate regulatory protein AlgP | -3.47 | 0.001636 |
| W6R202 | BN5_4203 | 50S ribosomal protein L28 | -3.48 | 0.011133 |
| W6R3E0 | BN5_2326 | 50S ribosomal protein L20 | -3.77 | 0.00457 |
| W6RJP7 | BN5_3492 | Sulfate adenylyltransferase subunit 2 (EC 2.7.7.4) (ATP-sulfurylase small subunit) (Sulfate adenylate transferase) (SAT) | -3.78 | 8.76E-05 |
| W6RLE3 | BN5_4165 | Antioxidant, AhpC/Tsa family (EC 1.11.1.15) | -4.08 | 0.003468 |
| W6QXZ3 | BN5_0422 | 50S ribosomal protein L31 | -4.12 | 0.034866 |
| W6QZG6 | BN5_3683 | 30S ribosomal protein S14 | -4.32 | 0.008732 |
| W6R788 | BN5_3674 | 30S ribosomal protein S13 | -4.33 | 0.011453 |
| W6RE84 | BN5_1603 | Glutamate dehydrogenase | -11.42 | 0.000253 |
| W6RLN9 | BN5_4250 | NAD(P)(+) transhydrogenase (AB-specific) (EC 1.6.1.2) | -12.01 | 0.006448 |
| W6QRT0 | BN5_0092 | Cytochrome c oxidase subunit 2 (EC 1.9.3.1) | CNA | - |
| W6RA38 | BN5_0113 | N-acetylmuramoyl-L-alanine amidase (EC 3.5.1.28) | CNA | - |
| W6QQG6 | BN5_0126 | Glycolate oxidase iron-sulfur subunit | CNA | - |
| W6RA91 | BN5_0128 | Glycolate oxidase, subunit GlcD (EC 1.1.3.15) | CNA | - |
| W6RAA1 | BN5_0139 | ABC transporter, solute-binding component | CNA | - |

| | | | | |
|---|---|---|---|---|
| W6QQI9 | BN5_0142 | ABC transporter, ATP-binding component (EC 3.6.3.21) | CNA | - |
| W6QPN4 | BN5_0291 | ATP-binding protein of ABC transporter | CNA | - |
| W6RAQ9 | BN5_0315 | Protein-glutamate methylesterase CheB (EC 3.1.1.61) | CNA | - |
| W6QQZ8 | BN5_0328 | Dihydropyrimidine dehydrogenase (EC 1.3.1.2) | CNA | - |
| W6QSH6 | BN5_0329 | Glutamate synthase (EC 1.4.1.13) | CNA | - |
| W6QXQ0 | BN5_0332 | N-carbamoyl-L-amino acid amidohydrolase (EC 3.5.1.87) | CNA | - |
| W6QSI1 | BN5_0334 | Methyl-accepting chemotaxis protein I | CNA | - |
| W6QXX9 | BN5_0412 | Polyhydroxyalkanoate synthase, class II (EC 2.3.1.-) | CNA | - |
| W6QRB2 | BN5_0438 | Fis family transcriptional regulator | CNA | - |
| W6QST4 | BN5_0439 | ABC-type transporter periplasmic component protein | CNA | - |
| W6QQ44 | BN5_0451 | UPF0061 protein BN5_0451 | CNA | - |
| W6QYC6 | BN5_0544 | Hypotetical protein | CNA | - |
| W6RBC9 | BN5_0552 | Urease accessory protein UreG | CNA | - |
| W6QQF7 | BN5_0578 | Urease subunit alpha (EC 3.5.1.5) (Urea amidohydrolase subunit alpha) | CNA | - |
| W6QYH5 | BN5_0589 | ABC transporter related | CNA | - |
| W6QRS0 | BN5_0590 | ABC transporter related | CNA | - |
| W6RBH5 | BN5_0592 | Inner-membrane translocator | CNA | - |
| W6QS16 | BN5_0683 | Phosphoenolpyruvate-protein phosphotransferase (EC 2.7.3.9) | CNA | - |
| W6RBV5 | BN5_0715 | Oligopeptide/dipeptide ABC transporter, ATPase subunit | CNA | - |
| W6QR22 | BN5_0761 | Acyl-CoA dehydrogenase domain-containing protein | CNA | - |
| W6RBZ4 | BN5_0770 | Uncharacterized protein | CNA | - |
| W6QTV4 | BN5_0784 | Pseudouridine synthase (EC 5.4.99.-) | CNA | - |
| W6RC47 | BN5_0835 | ABC transporter, ATP-binding protein (EC 3.6.3.17) | CNA | - |
| W6QZ59 | BN5_0852 | Pyridoxamine 5'-phosphate oxidase-related, FMN-binding (EC 1.4.3.5) | CNA | - |
| W6QZK3 | BN5_1009 | Putative ATP-binding component of a transport system | CNA | - |
| H9N5E0 | BN5_1630 | Sigma-54-dependent transcriptional regulator (Transcriptional regulator) | CNA | - |
| H9N5E1 | BN5_1632 | Nitrilase (EC 3.5.5.7) | CNA | - |
| H9N5E5 | BN5_1635 | AIR synthase related protein domain protein (AIR synthase-like protein) | CNA | - |
| H9N5D8 | BN5_1637 | FAD dependent oxidoreductase (FAD-dependent oxidoreductase) | CNA | - |
| W6REF7 | BN5_1648 | Phospholipase D/Transphosphatidylase (EC 2.7.8.-) | CNA | - |
| W6QUX8 | BN5_1661 | RecBCD enzyme subunit RecD (EC 3.1.11.5) (Exonuclease V subunit RecD) (ExoV subunit RecD) (Helicase/nuclease RecBCD subunit RecD) | CNA | - |
| W6QTY0 | BN5_1774 | Methyl-accepting chemotaxis protein tlpB | CNA | - |
| W6QVA7 | BN5_1826 | Acyl-CoA dehydrogenase family protein | CNA | - |
| W6QU68 | BN5_1874 | Replication-associated recombination protein A | CNA | - |
| W6RF21 | BN5_1905 | Histidinol-phosphate aminotransferase (EC 2.6.1.9) (Imidazole acetol-phosphate transaminase) | CNA | - |
| W6RF39 | BN5_1925 | Aliphatic nitrilase (EC 3.5.5.7) | CNA | - |
| W6QXA8 | BN5_2056 | Putative CheW protein (EC 2.7.13.3) | CNA | - |
| W6QUU3 | BN5_2114 | Nitrate transporter periplasmic component | CNA | - |
| W6RFM9 | BN5_2123 | Assimilatory nitrite reductase (Subunit) (EC 1.7.1.4) | CNA | - |
| W6R2U2 | BN5_2125 | Nitrate reductase (EC 1.7.99.4) | CNA | - |
| W6QVA8 | BN5_2234 | Amidotransferase | CNA | - |
| W6QVB9 | BN5_2244 | Transglutaminase domain-containing protein | CNA | - |
| W6QWF9 | BN5_2256 | Putative two-component response regulator | CNA | - |
| W6RG67 | BN5_2293 | UPF0502 protein BN5_2293 | CNA | - |
| W6R3G9 | BN5_2367 | Extracellular solute-binding protein | CNA | - |
| W6QWV0 | BN5_2413 | GntR family transcriptional regulator | CNA | - |

| | | | | |
|---|---|---|---|---|
| W6QYC5 | BN5_2449 | Cytochrome c oxidase accessory protein, cbb3-type, CcoG | CNA | - |
| W6R3S8 | BN5_2477 | Nitroreductase | CNA | - |
| W6QX60 | BN5_2518 | Metallo-beta-lactamase family protein (EC 3.1.2.6) | CNA | - |
| W6QZD5 | BN5_2808 | S-formylglutathione hydrolase (EC 3.1.2.12) | CNA | - |
| W6QY32 | BN5_2822 | Acyl-[acyl-carrier-protein]--UDP-N-acetylglucosamine O-acyltransferase (UDP-N-acetylglucosamine acyltransferase) (EC 2.3.1.129) | CNA | - |
| W6QYG7 | BN5_2962 | Penicillin amidase family protein (EC 3.5.1.11) | CNA | - |
| W6QZX4 | BN5_2993 | Uracil-DNA glycosylase (UDG) (EC 3.2.2.27) | CNA | - |
| W6QZY9 | BN5_3008 | 3-hydroxyacyl-CoA-acyl carrier protein transferase (EC 2.4.1.-) | CNA | - |
| W6QYM4 | BN5_3018 | Serine hydroxymethyltransferase (SHMT) (Serine methylase) (EC 2.1.2.1) | CNA | - |
| W6R002 | BN5_3024 | Peptidase M14, carboxypeptidase A | CNA | - |
| W6QXL2 | BN5_3048 | 6-phosphogluconate dehydratase (EC 4.2.1.12) | CNA | - |
| W6R5J7 | BN5_3085 | Holliday junction ATP-dependent DNA helicase RuvB (EC 3.6.4.12) | CNA | - |
| W6QXS7 | BN5_3119 | Putative quercetin 2,3-dioxygenase PA3240 (EC 1.13.11.24) | CNA | - |
| W6R097 | BN5_3122 | Probable septum site-determining protein MinC | CNA | - |
| W6QZ82 | BN5_3191 | Pyrimidine biosynthesis enzyme THI13 | CNA | - |
| W6R0K2 | BN5_3192 | 3-ketoacyl-(Acyl-carrier-protein) reductase (EC 1.1.1.100) | CNA | - |
| W6RJ07 | BN5_3193 | Dihydroorotase (EC 3.5.2.3) | CNA | - |
| W6R5Z8 | BN5_3200 | Uncharacterized protein | CNA | - |
| W6R622 | BN5_3235 | Periplasmic oligopeptide-binding protein | CNA | - |
| W6QZC3 | BN5_3236 | Acetamidase/formamidase (EC 3.5.1.4) | CNA | - |
| W6R0U1 | BN5_3308 | Butyryl-CoA:acetate CoA transferase (EC 2.8.3.5) | CNA | - |
| W6RJF8 | BN5_3397 | PhoH family protein | CNA | - |
| W6R6I9 | BN5_3444 | Response regulator receiver modulated diguanylate cyclase (EC 3.1.1.61) | CNA | - |
| W6R6Q1 | BN5_3509 | ABC transporter related (EC 3.6.3.27) | CNA | - |
| W6QZ64 | BN5_3598 | GCN5-related N-acetyltransferase (EC 2.3.1.48) | CNA | - |
| W6R709 | BN5_3604 | Oxidoreductase FAD-binding region (EC 1.17.1.1) | CNA | - |
| W6QZR6 | BN5_3762 | Polyamine-transporting ATPase (EC 3.6.3.31) | CNA | - |
| W6R822 | BN5_4009 | Polyamine-transporting ATPase (EC 3.6.3.31) | CNA | - |
| W6R1Q3 | BN5_4128 | 1,4-beta-D-xylan xylohydrolase (EC 3.2.1.37) | CNA | - |
| W6R1R3 | BN5_4138 | dTDP-4-dehydrorhamnose reductase (EC 1.1.1.133) | CNA | - |
| W6R0Y2 | BN5_4196 | Uncharacterized protein | CNA | - |
| W6R277 | BN5_4268 | Breakpoint cluster region protein (EC 2.7.11.1) | CNA | - |
| W6R2I3 | BN5_4383 | Uncharacterized protein PYRAB12460 | CNA | - |
| W6R420 | BN5_4384 | Putative oxidoreductase (EC 1.1.1.-) | CNA | - |
| W6QQE5 | BN5_0116 | Phosphate transport system permease protein | N | - |
| W6QS38 | BN5_0169 | Xanthine phosphoribosyltransferase (XPRTase) (EC 2.4.2.22) | N | - |
| W6QPC3 | BN5_0171 | ATP-dependent DNA helicase Rep (EC 3.6.4.12) | N | - |
| W6RAS2 | BN5_0335 | Cytochrome c | N | - |
| W6RBB8 | BN5_0542 | UPF0313 protein BN5_0542 | N | - |
| W6QS04 | BN5_0663 | Type II secretion system protein | N | - |
| W6QTM2 | BN5_0694 | TonB-dependent siderophore receptor | N | - |
| W6QRB3 | BN5_0856 | 50S ribosomal protein L27 | N | - |
| W6QSN9 | BN5_0908 | DNA repair protein RecN (Recombination protein N) | N | - |
| W6QU71 | BN5_0909 | Protein GrpE (HSP-70 cofactor) | N | - |
| W6QRJ2 | BN5_0933 | 30S ribosomal protein S15 | N | - |
| W6QRY4 | BN5_1103 | Uncharacterized protein | N | - |

| | | | | |
|---|---|---|---|---|
| W6R0C8 | BN5_1270 | Putative peptidase (EC 3.4.-.-) | N | - |
| W6RDQ8 | BN5_1420 | Uncharacterized protein yggL | N | - |
| W6QUC9 | BN5_1488 | Arginine/ornithine transport protein AotP (EC 3.6.3.-) | N | - |
| W6QTH8 | BN5_1644 | Uncharacterized protein | N | - |
| W6R1R4 | BN5_1740 | Cytosine-specific methyltransferase (EC 2.1.1.37) | N | - |
| W6R378 | BN5_2250 | Uncharacterized protein | N | - |
| W6QWF4 | BN5_2251 | Coenzyme PQQ synthesis protein F (EC 3.4.99.-) | N | - |
| W6R3E6 | BN5_2337 | Coenzyme PQQ synthesis protein E (Pyrroloquinoline quinone biosynthesis protein E) | N | - |
| W6RGJ0 | BN5_2450 | Cytochrome c oxidase accessory protein CcoH | N | - |
| W6QW61 | BN5_2565 | Chromosome partition protein Smc | N | - |
| W6RI39 | BN5_2899 | Ribonuclease 3 (EC 3.1.26.3) (Ribonuclease III) (RNase III) | N | - |
| W6QYQ0 | BN5_3050 | Methylglyoxal synthase (MGS) (EC 4.2.3.3) | N | - |
| W6RIW6 | BN5_3173 | Uncharacterized protein | N | - |
| W6RIZ3 | BN5_3183 | ISPpu14, transposase Orf3 | N | - |
| W6R634 | BN5_3255 | Uncharacterized protein | N | - |
| W6R0Q7 | BN5_3267 | Iron-sulfur cluster assembly scaffold protein IscU | N | - |
| W6R0R5 | BN5_3277 | Queuine tRNA-ribosyltransferase (EC 2.4.2.29) (Guanine insertion enzyme) (tRNA-guanine transglycosylase) | N | - |
| W6R0S0 | BN5_3283 | Permease YjgP/YjgQ family protein | N | - |
| W6RJH8 | BN5_3427 | Peptidyl-prolyl cis-trans isomerase (EC 5.2.1.8) | N | - |
| W6QZZ6 | BN5_3480 | Cytochrome b | N | - |
| W6R6Z2 | BN5_3589 | PAS/PAC sensor signal transduction histidine kinase | N | - |
| W6R775 | BN5_3664 | dTDP-4-dehydrorhamnose reductase (EC 1.1.1.133) | N | - |
| W6R7C9 | BN5_3704 | 50S ribosomal protein L7/L12 | N | - |
| W6R290 | BN5_3816 | Uncharacterized protein | N | - |
| W6RKS8 | BN5_3917 | Biotin synthase (EC 2.8.1.6) | N | - |
| W6R047 | BN5_3928 | DNA polymerase III, epsilon subunit (EC 2.7.7.7) | N | - |
| W6RKV5 | BN5_3957 | tRNA sulfurtransferase (EC 2.8.1.4) (Sulfur carrier protein ThiS sulfurtransferase) (Thiamine biosynthesis protein ThiI) (tRNA 4-thiouridine synthase) | N | - |
| W6R8C8 | BN5_4121 | Integrase | N | - |
| W6R108 | BN5_4201 | Chemotaxis response regulator protein-glutamate methylesterase 1 (EC 3.1.1.61) | N | - |
| W6R8S5 | BN5_4227 | Uncharacterized protein | N | - |
| W6R959 | BN5_4392 | Probable tonB-dependent receptor yncD | N | - |
| W6RM88 | BN5_4500 | ATP synthase subunit delta (ATP synthase F(1) sector subunit delta) (F-type ATPase subunit delta) (F-ATPase subunit delta) | N | - |

**Table S2**. Proteins differentially expressed in *P. pseudoalcaligenes* CECT5344 in response to the cyanide-containing jewelry residue using ammonium as control. Proteins significantly up-regulated (fold change ≥ 2) and down-regulated (fold change ≤ 2) are shown. “R” or “N” indicates that proteins were detected exclusively in cells grown with the residue or ammonium, respectively.

| Uniprot ID | Gene locus | Protein Name | Fold Change R/N | *p* value |
|---|---|---|---|---|
| W6QYV6 | BN5_0702 | Heavy metal translocating P-type ATPase (EC 3.6.3.4) | 453.86 | 0.004078 |
| W6R260 | BN5_1907 | 4-hydroxy-tetrahydrodipicolinate synthase (HTPA synthase) (EC 4.3.3.7) | 155.94 | 9.68E-06 |
| W6R265 | BN5_1912 | Nitrilase (EC 3.5.5.1) | 147.93 | 0.001059 |
| W6QTM2 | BN5_0694 | TonB-dependent siderophore receptor | 15.06 | 0.011624 |
| W6R1F1 | BN5_4010 | Spermidine/putrescine-binding periplasmic protein 1 | 11.32 | 0.028131 |
| W6QQH3 | BN5_0593 | ABC-type branched-chain amino acid transport systems periplasmic component-like protein | 8.70 | 0.000488 |
| W6QQC6 | BN5_0543 | Transglutaminase domain-containing protein | 8.11 | 4.93E-05 |
| W6QWF0 | BN5_2246 | Uncharacterized protein | 7.09 | 0.000742 |
| W6R0L4 | BN5_3207 | Basic membrane protein A2 Immunodominant antigen P39 | 6.82 | 0.013247 |
| W6R374 | BN5_2245 | Uncharacterized protein | 6.48 | 0.025679 |
| W6QXC5 | BN5_0151 | Flavin monoamine oxidase-related protein (EC 1.4.3.4) | 5.94 | 0.007128 |
| W6RKV8 | BN5_3962 | Nitrogen regulation protein NR(I) | 5.51 | 0.016608 |
| W6R077 | BN5_3958 | Glutamine synthetase (EC 6.3.1.2) | 5.36 | 0.000139 |
| W6RAC3 | BN5_0149 | Putative endoribonuclease L-PSP | 5.32 | 0.011032 |
| W6RC44 | BN5_0830 | Basic membrane lipoprotein | 4.97 | 0.005558 |
| W6QY03 | BN5_0432 | Type IV pilus assembly protein PilQ | 4.89 | 0.000288 |
| W6QY43 | BN5_2354 | ABC transporter periplasmic protein | 4.85 | 0.002581 |
| W6QQS0 | BN5_0248 | Binding protein component of ABC iron transporter | 4.37 | 0.003051 |
| W6QUE9 | BN5_1006 | Putative amino-acid ABC transporter-binding protein | 4.24 | 0.000287 |
| W6QZT0 | BN5_2953 | Outer membrane porin F | 4.17 | 0.000194 |
| W6QPA6 | BN5_0150 | Cytochrome C6 | 4.00 | 0.017826 |
| W6RFI0 | BN5_2077 | Uncharacterized protein | 3.94 | 0.002635 |
| W6QYV0 | BN5_3096 | Porin D (EC 3.4.21.-) | 3.73 | 0.002437 |
| W6QTP6 | BN5_0719 | Extracellular solute-binding protein | 3.68 | 0.002009 |
| W6QTN7 | BN5_1245 | Soluble aldose sugar dehydrogenase yliI (EC 1.1.5.-) | 3.64 | 0.003355 |
| W6QXK4 | BN5_2157 | Outer membrane lipoprotein OprI | 3.19 | 0.002117 |
| W6RDL1 | BN5_1358 | Probable malate:quinone oxidoreductase (EC 1.1.5.4) (MQO) (Malate dehydrogenase [quinone]) | 2.99 | 0.010765 |
| W6QS11 | BN5_0127 | Glycolate oxidase FAD binding subunit (EC 1.1.3.15) | 2.98 | 0.002118 |
| W6RE08 | BN5_1524 | Fumarate hydratase class II (Fumarase C) (EC 4.2.1.2) | 2.90 | 0.001361 |
| W6R2G4 | BN5_3896 | NADPH:quinone oxidoreductase 1 (EC 1.6.5.2) | 2.85 | 0.003237 |
| W6RL49 | BN5_4062 | Uncharacterized protein | 2.79 | 0.000581 |
| W6QPR3 | BN5_0321 | UPF0312 protein BN5_0321 | 2.76 | 0.003084 |
| W6RKJ4 | BN5_3761 | Extracellular solute-binding protein | 2.68 | 0.002496 |
| W6RCF1 | BN5_0962 | Cyclic nucleotide-binding protein | 2.64 | 0.004196 |
| W6QW62 | BN5_1647 | ATP-dependent Clp protease ATP-binding subunit ClpC (EC 3.1.21.-) | 2.57 | 0.000587 |
| W6QR96 | BN5_0836 | Short chain dehydrogenase | 2.55 | 0.011163 |
| W6QWW6 | BN5_1894 | Uncharacterized protein | 2.50 | 0.003231 |
| W6QWA0 | BN5_1657 | Lipoprotein heavy metal/multidrug efflux protein | 2.46 | 0.025461 |
| W6RFJ3 | BN5_2092 | Copper resistance B | 2.34 | 0.013394 |
| W6QUT3 | BN5_1646 | Small heat shock protein | 2.31 | 6.07E-05 |

| | | | | |
|---|---|---|---|---|
| W6R2R6 | BN5_4453 | Uncharacterized protein | 2.29 | 0.0112 |
| W6QSM0 | BN5_0374 | Substrate-binding region of ABC-type glycine betaine transport system | 2.28 | 0.005657 |
| W6QVZ5 | BN5_2095 | Outer membrane porin, OprD family | 2.25 | 0.001812 |
| W6R5A3 | BN5_3001 | 3-hydroxyisobutyrate dehydrogenase (HIBADH) (EC 1.1.1.31) | 2.23 | 0.006138 |
| W6QTK8 | BN5_1649 | ATP-dependent zinc metalloprotease FtsH (EC 3.4.24.-) | 2.20 | 0.00032 |
| W6R8G1 | BN5_4152 | Porin D (EC 3.4.21.-) | 2.17 | 0.017064 |
| W6R1W7 | BN5_1805 | Uncharacterized protein | 2.16 | 0.009127 |
| W6R782 | BN5_3669 | Catalase-peroxidase (CP) (EC 1.11.1.21) (Peroxidase/catalase) | 2.15 | 0.000213 |
| W6QQ88 | BN5_0496 | Outer membrane channel protein | 2.13 | 0.011837 |
| W6RB40 | BN5_0465 | Pyruvate dehydrogenase E1 component (EC 1.2.4.1) | 2.12 | 0.002093 |
| W6R0J6 | BN5_4079 | Membrane-bound lytic murein transglycosylase F | 2.10 | 0.012521 |
| W6R8V0 | BN5_4267 | Putative virulence effector protein | 2.09 | 0.017482 |
| W6R5D0 | BN5_3027 | ABC-type Fe3+ transport system periplasmic component-like protein | 2.08 | 0.028457 |
| W6QQ57 | BN5_0466 | Glutamate-ammonia-ligase adenylyltransferase (EC 2.7.7.42) (Glutamine-synthase adenylyltransferase) (ATase) ([Glutamate--ammonia-ligase] adenylyltransferase) | 2.06 | 0.002189 |
| W6RK30 | BN5_3612 | Putative periplasmic binding protein | 2.06 | 0.015052 |
| W6QYW7 | BN5_3493 | GTP cyclohydrolase 1 type 2 homolog | 2.05 | 0.014667 |
| W6RKK5 | BN5_3776 | Putative serine protein kinase, PrkA | 2.03 | 6E-06 |
| W6QZ51 | BN5_0842 | Beta-alanine-pyruvate transaminase (EC 2.6.1.18) | 2.01 | 0.004145 |
| W6R3F0 | BN5_2342 | PQQ-containing dehydrogenase | 2.01 | 0.008395 |
| W6R2H7 | BN5_2024 | ATP-dependent RNA helicase DeaD (EC 3.6.4.13) (Cold-shock DEAD box protein A) | -2.06 | 0.006563 |
| W6QZM4 | BN5_3703 | DNA-directed RNA polymerase subunit beta (RNAP subunit beta) (EC 2.7.7.6) (RNA polymerase subunit beta) (Transcriptase subunit beta) | -2.06 | 0.001582 |
| W6R6R3 | BN5_3519 | Leucine--tRNA ligase (EC 6.1.1.4) (Leucyl-tRNA synthetase) (LeuRS) | -2.09 | 0.031037 |
| W6QQ48 | BN5_0456 | Ribosomal RNA large subunit methyltransferase J (EC 2.1.1.266) (23S rRNA (adenine(2030)-N6)-methyltransferase) (23S rRNA m6A2030 methyltransferase) | -2.11 | 0.004557 |
| W6R5P9 | BN5_3130 | Lon protease (EC 3.4.21.53) (ATP-dependent protease La) | -2.11 | 0.009075 |
| W6QQH7 | BN5_0598 | Phosphoribosylamine--glycine ligase (EC 6.3.4.13) (GARS) (Glycinamide ribonucleotide synthetase) (Phosphoribosylglycinamide synthetase) | -2.11 | 0.014321 |
| W6QP53 | BN5_0119 | Phosphate-specific transport system accessory protein PhoU | -2.12 | 0.027154 |
| W6QZF3 | BN5_3673 | 30S ribosomal protein S11 | -2.14 | 0.007446 |
| W6R197 | BN5_3491 | Adenylyl-sulfate kinase (EC 2.7.1.25) (EC 2.7.7.4) (APS kinase) (ATP adenosine-5'-phosphosulfate 3'-phosphotransferase) (ATP-sulfurylase large subunit) (Adenosine-5'-phosphosulfate kinase) (Sulfate adenylate transferase) (SAT) (Sulfate adenylyltransferase subunit 1) | -2.14 | 0.010155 |
| W6QUD1 | BN5_0981 | Chromosome segregation ATPase | -2.15 | 0.00345 |
| W6RJP2 | BN5_3487 | Cell division protein ZapE (Z ring-associated protein ZapE) | -2.20 | 0.013662 |
| W6R0I3 | BN5_3670 | 50S ribosomal protein L17 | -2.23 | 0.005529 |
| W6QV37 | BN5_1751 | Lon protease (EC 3.4.21.53) (ATP-dependent protease La) | -2.25 | 0.011532 |
| W6RKA2 | BN5_3672 | 30S ribosomal protein S4 | -2.27 | 0.001459 |
| W6R2W1 | BN5_4498 | ATP synthase gamma chain (ATP synthase F1 sector gamma subunit) (F-ATPase gamma subunit) | -2.29 | 0.004028 |
| W6QYJ1 | BN5_0604 | Acetyl-CoA carboxylase biotin carboxylase subunit (EC 6.4.1.2) | -2.29 | 0.000733 |
| W6QXF0 | BN5_0187 | Diaminopimelate epimerase (DAP epimerase) (EC 5.1.1.7) | -2.30 | 0.006955 |
| W6RDN8 | BN5_1393 | Glyceraldehyde-3-phosphate dehydrogenase (EC 1.2.1.-) | -2.32 | 0.005137 |
| W6QQ64 | BN5_0001 | Chromosomal replication initiator protein DnaA | -2.34 | 0.015373 |
| W6QTT2 | BN5_1291 | Ribonucleoside-diphosphate reductase (EC 1.17.4.1) | -2.36 | 0.000523 |
| W6RKW1 | BN5_3967 | Chaperone protein clpB | -2.39 | 0.004408 |

| | | | | |
|---|---|---|---|---|
| W6QXP5 | BN5_0322 | ATP-dependent RNA helicase RhlE (EC 3.6.4.13) | -2.43 | 0.000424 |
| W6QUZ6 | BN5_1206 | Pyruvate kinase (EC 2.7.1.40) | -2.45 | 0.011678 |
| W6QYI6 | BN5_0599 | Bifunctional purine biosynthesis protein PurH [Includes: IMP cyclohydrolase (EC 3.5.4.10) (ATIC) (Inosinicase) (IMP synthase); Phosphoribosylaminoimidazolecarboxamide formyltransferase (EC 2.1.2.3) (AICAR transformylase)] | -2.50 | 0.006144 |
| W6QQU7 | BN5_0278 | Resolvase | -2.52 | 0.000922 |
| W6REY3 | BN5_1853 | Isocitrate lyase (EC 4.1.3.1) | -2.59 | 0.00341 |
| W6REZ0 | BN5_1863 | Isocitrate dehydrogenase (EC 1.1.1.42) | 2.60 | 0.001035 |
| W6R034 | BN5_1149 | Formyltetrahydrofolate deformylase (EC 3.5.1.10) (Formyl-FH(4) hydrolase) | -2.64 | 0.007736 |
| W6R2X2 | BN5_4508 | tRNA uridine 5-carboxymethylaminomethyl modification enzyme MnmG (Glucose-inhibited division protein A) | -2.71 | 0.001106 |
| W6R0J3 | BN5_3680 | 50S ribosomal protein L18 | -2.71 | 0.024036 |
| W6QZN2 | BN5_1039 | 2-isopropylmalate synthase (EC 2.3.3.13) (Alpha-IPM synthase) (Alpha-isopropylmalate synthase) | -2.71 | 0.022605 |
| W6R6R8 | BN5_3524 | Transcriptional regulator, XRE family | -2.71 | 0.006106 |
| W6R473 | BN5_4434 | Peptide methionine sulfoxide reductase MsrA (Protein-methionine-S-oxide reductase) (EC 1.8.4.11) (Peptide-methionine (S)-S-oxide reductase) (Peptide Met(O) reductase) | -2.73 | 0.001245 |
| W6QSV0 | BN5_0970 | Acetolactate synthase (EC 2.2.1.6) | -2.74 | 0.008406 |
| W6QS70 | BN5_0214 | Putative ABC transporter ATP-binding protein yheS | -2.79 | 0.01661 |
| W6R168 | BN5_3925 | Malate synthase G (EC 2.3.3.9) | -2.82 | 0.000802 |
| W6QYV7 | BN5_3483 | 50S ribosomal protein L13 | -2.83 | 0.000383 |
| W6QUZ9 | BN5_1686 | UDP-N-acetyl-D-mannosamine dehydrogenase (EC 1.1.1.-) | -3.01 | 0.001474 |
| W6QSW2 | BN5_0985 | Saccharopine dehydrogenase (EC 1.5.1.7) | -3.02 | 0.001183 |
| W6R1N1 | BN5_1685 | UDP-N-acetylglucosamine 2-epimerase (EC 5.1.3.14) | -3.09 | 0.000118 |
| W6RC63 | BN5_0860 | Probable malate:quinone oxidoreductase (EC 1.1.5.4) (MQO) (Malate dehydrogenase [quinone]) | -3.09 | 0.003636 |
| W6QUJ2 | BN5_1560 | GTP pyrophosphokinase (EC 2.7.6.5) | -3.12 | 0.01887 |
| W6QT13 | BN5_1030 | Uncharacterized protein | -3.25 | 0.000822 |
| W6R3W8 | BN5_4334 | Import inner membrane translocase, subunit Tim44 | -3.29 | 0.001146 |
| W6QZF8 | BN5_3678 | 50S ribosomal protein L30 | -3.54 | 0.00159 |
| W6RJL0 | BN5_3457 | Cell division protein FtsZ | -3.64 | 0.000549 |
| W6R3I5 | BN5_2387 | 3-isopropylmalate dehydratase small subunit (EC 4.2.1.33) (Alpha-IPM isomerase) (IPMI) (Isopropylmalate isomerase) | -3.77 | 0.001181 |
| W6R0E8 | BN5_1290 | Ribonucleoside-diphosphate reductase subunit beta (EC 1.17.4.1) | -3.85 | 0.000317 |
| W6QNV1 | BN5_0004 | DNA gyrase subunit B (EC 5.99.1.3) | -4.20 | 0.003094 |
| W6RC60 | BN5_0855 | 50S ribosomal protein L21 | -4.78 | 0.001448 |
| W6R202 | BN5_4203 | 50S ribosomal protein L28 | -5.42 | 0.002343 |
| W6RKD3 | BN5_3687 | 30S ribosomal protein S17 | -5.84 | 0.000183 |
| W6QWS7 | BN5_2388 | 3-isopropylmalate dehydratase large subunit (EC 4.2.1.33) (Alpha-IPM isomerase) (IPMI) (Isopropylmalate isomerase) | -5.97 | 0.000421 |
| W6RLE3 | BN5_4165 | Antioxidant, AhpC/Tsa family (EC 1.11.1.15) | -6.06 | 0.004526 |
| W6RA44 | BN5_0118 | Phosphate import ATP-binding protein PstB (EC 3.6.3.27) (ABC phosphate transporter) (Phosphate-transporting ATPase) | -7.00 | 0.007325 |
| W6RLN9 | BN5_4250 | NAD(P)(+) transhydrogenase (AB-specific) (EC 1.6.1.2) | -7.53 | 0.001154 |
| W6RE84 | BN5_1603 | Glutamate dehydrogenase | -8.03 | 0.000431 |
| W6RJP7 | BN5_3492 | Sulfate adenylyltransferase subunit 2 (EC 2.7.7.4) (ATP-sulfurylase small subunit) (Sulfate adenylate transferase) (SAT) | -15.46 | 2.67E-05 |
| W6QWV7 | BN5_0025 | Ribosomal RNA small subunit methyltransferase B (EC 2.1.1.-) | N | - |
| W6QX52 | BN5_0105 | Probable GTP-binding protein EngB | N | - |
| W6QQE5 | BN5_0116 | Phosphate transport system permease protein | N | - |

| | | | | |
|---|---|---|---|---|
| W6QQK7 | BN5_0168 | Cytochrome c5-like protein | N | - |
| W6QPC3 | BN5_0171 | ATP-dependent DNA helicase Rep (EC 3.6.4.12) | N | - |
| W6RAQ0 | BN5_0310 | Response regulator receiver protein | N | - |
| W6QR20 | BN5_0348 | Lipoprotein, putative | N | - |
| W6QSW5 | BN5_0469 | Lipopolysaccharide heptosyltransferase I (EC 2.4.-.-) | N | - |
| W6QQ70 | BN5_0476 | Glycosyl transferase, family 2 (EC 2.4.-.-) | N | - |
| W6QY48 | BN5_0477 | Uncharacterized protein | N | - |
| W6RB57 | BN5_0480 | Capsule polysaccharide export protein | N | - |
| W6RBB8 | BN5_0542 | UPF0313 protein BN5_0542 | N | - |
| W6QQD1 | BN5_0548 | Cytokinin riboside 5'-monophosphate phosphoribohydrolase (EC 3.2.2.n1) | N | - |
| W6QQV2 | BN5_0691 | Peptide chain release factor 3 (RF-3) | N | - |
| W6RBV0 | BN5_0710 | tRNA/tmRNA (uracil-C(5))-methyltransferase (EC 2.1.1.-) (EC 2.1.1.35) (tRNA (uracil(54)-C(5))-methyltransferase) (tRNA(m5U54)-methyltransferase) (RUMT) (tmRNA (uracil(341)-C(5))-methyltransferase) | N | - |
| W6QS68 | BN5_0743 | CBS domain-containing protein | N | - |
| W6RBX9 | BN5_0745 | PhoH family protein | N | - |
| W6QTY1 | BN5_0814 | Hydrophobe/amphiphile efflux-1 (HAE1) family protein | N | - |
| W6QRB3 | BN5_0856 | 50S ribosomal protein L27 | N | - |
| W6QSI8 | BN5_0858 | Glutamate 5-kinase (EC 2.7.2.11) (Gamma-glutamyl kinase) (GK) | N | - |
| W6QRE4 | BN5_0886 | Beta-lactamase domain-containing protein | N | - |
| W6QRF6 | BN5_0896 | Nitroreductase (EC 1.-.-.-) | N | - |
| W6QSN9 | BN5_0908 | DNA repair protein RecN (Recombination protein N) | N | - |
| W6QRI7 | BN5_0928 | Ribosome maturation factor RimP | N | - |
| W6QRJ2 | BN5_0933 | 30S ribosomal protein S15 | N | - |
| W6QUH1 | BN5_1031 | Adenine-specific DNA-methyltransferase (EC 2.1.1.72) | N | - |
| W6QRX5 | BN5_1088 | Oxidoreductase, GMC family (EC 1.1.99.1) | N | - |
| W6QSC0 | BN5_1238 | Phosphonates import ATP-binding protein PhnC (EC 3.6.3.28) | N | - |
| W6R0C8 | BN5_1270 | Putative peptidase (EC 3.4.-.-) | N | - |
| W6RDG8 | BN5_1303 | Methyl-accepting chemotaxis serine transducer | N | - |
| W6R0H3 | BN5_1320 | CAIB/BAIF family protein (EC 2.8.3.16) | N | - |
| W6R0T3 | BN5_1432 | Glycerophosphoryl diester phosphodiesterase, putative (EC 3.1.4.46) | N | - |
| W6RDS2 | BN5_1435 | Lipoprotein-releasing system transmembrane protein lolC | N | - |
| W6RDT9 | BN5_1455 | Malonyl CoA-acyl carrier protein transacylase (EC 2.3.1.39) | N | - |
| W6QUC9 | BN5_1488 | Arginine/ornithine transport protein AotP (EC 3.6.3.-) | N | - |
| W6RDY8 | BN5_1500 | Carbon storage regulator homolog | N | - |
| W6QVX3 | BN5_1543 | Ribosomal protein S12 methylthiotransferase RimO (S12 MTTase) (S12 methylthiotransferase) (EC 2.8.4.4) (Ribosomal protein S12 (aspartate-C(3))-methylthiotransferase) (Ribosome maturation factor RimO) | N | - |
| W6QTH8 | BN5_1644 | Uncharacterized protein | N | - |
| W6R1R4 | BN5_1740 | Cytosine-specific methyltransferase (EC 2.1.1.37) | N | - |
| W6R238 | BN5_1886 | Response regulator in two-component regulatory system | N | - |
| W6R327 | BN5_2180 | Succinate dehydrogenase hydrophobic membrane anchor subunit | N | - |
| W6R360 | BN5_2225 | Peptide ABC transporter, putative ATP-binding protein (EC 3.6.3.25) | N | - |
| W6QWF4 | BN5_2251 | Coenzyme PQQ synthesis protein F (EC 3.4.99.-) | N | - |
| W6QXV6 | BN5_2252 | tRNA-dihydrouridine(20/20a) synthase (EC 1.3.1.-) (EC 1.3.1.91) (U20-specific dihydrouridine synthase) (U20-specific Dus) (tRNA-dihydrouridine synthase A) | N | - |
| W6RG60 | BN5_2283 | Uncharacterized protein | N | - |

| | | | | |
|---|---|---|---|---|
| W6R3E6 | BN5_2337 | Coenzyme PQQ synthesis protein E (Pyrroloquinoline quinone biosynthesis protein E) | N | - |
| W6QXK9 | BN5_2657 | Nitrogen regulation protein NR(I) | N | - |
| W6R4M0 | BN5_2738 | Uncharacterized protein | N | - |
| W6QXW7 | BN5_2749 | 2,4-dienoyl-coa reductase FADH1, putative (EC 1.3.1.34) | N | - |
| W6QX50 | BN5_2875 | ATP-dependent helicase HrpA (EC 3.6.1.-) | N | - |
| W6RI39 | BN5_2899 | Ribonuclease 3 (EC 3.1.26.3) (Ribonuclease III) (RNase III) | N | - |
| W6QYQ0 | BN5_3050 | Methylglyoxal synthase (MGS) (EC 4.2.3.3) | N | - |
| W6QYS5 | BN5_3076 | 7-cyano-7-deazaguanine synthase (EC 6.3.4.20) (7-cyano-7-carbaguanine synthase) (PreQ(0) synthase) (Queuosine biosynthesis protein QueC) | N | - |
| W6RIZ3 | BN5_3183 | ISPpu14, transposase Orf3 | N | - |
| W6R634 | BN5_3255 | Uncharacterized protein | N | - |
| W6R0R5 | BN5_3277 | Queuine tRNA-ribosyltransferase (EC 2.4.2.29) (Guanine insertion enzyme) (tRNA-guanine transglycosylase) | N | - |
| W6R0S0 | BN5_3283 | Permease YjgP/YjgQ family protein | N | - |
| W6QZI3 | BN5_3307 | 3-oxoacid CoA-transferase (EC 2.8.3.5) | N | - |
| W6QYG6 | BN5_3315 | Beta-lactamase domain-containing protein | N | - |
| W6QYV2 | BN5_3478 | Glutathione S-transferase domain-containing protein (EC 2.5.1.18) | N | - |
| W6R6W3 | BN5_3564 | Lipoyl synthase (EC 2.8.1.8) (Lip-syn) (LS) (Lipoate synthase) (Lipoic acid synthase) (Sulfur insertion protein LipA) | N | - |
| W6R6Z2 | BN5_3589 | PAS/PAC sensor signal transduction histidine kinase | N | - |
| W6R1P1 | BN5_3646 | Riboflavin biosynthesis protein RibD | N | - |
| W6R775 | BN5_3664 | dTDP-4-dehydrorhamnose reductase (EC 1.1.1.133) | N | - |
| W6QZN4 | BN5_3717 | Bifunctional ligase/repressor BirA (Biotin operon repressor) (Biotin--[acetyl-CoA-carboxylase] ligase) (EC 6.3.4.15) (Biotin--protein ligase) (Biotin-[acetyl-CoA carboxylase] synthetase) | N | - |
| W6R290 | BN5_3816 | Uncharacterized protein | N | - |
| W6R2H4 | BN5_3906 | Acyl-CoA dehydrogenase domain-containing protein (EC 1.3.99.-) | N | - |
| W6RKS8 | BN5_3917 | Biotin synthase (EC 2.8.1.6) | N | - |
| W6R047 | BN5_3928 | DNA polymerase III, epsilon subunit (EC 2.7.7.7) | N | - |
| W6RKV5 | BN5_3957 | tRNA sulfurtransferase (EC 2.8.1.4) (Sulfur carrier protein ThiS sulfurtransferase) (Thiamine biosynthesis protein ThiI) (tRNA 4-thiouridine synthase) | N | - |
| W6R1C2 | BN5_3980 | tRNA (guanine-N(7)-)-methyltransferase (EC 2.1.1.33) (tRNA (guanine(46)-N(7))-methyltransferase) (tRNA(m7G46)-methyltransferase) | N | - |
| W6R0C3 | BN5_4008 | UPF0064 protein yccW | N | - |
| W6R1M3 | BN5_4097 | Poly(3-hydroxyalkanoate) synthetase (EC 3.1.1.-) | N | - |
| W6R8C8 | BN5_4121 | Integrase | N | - |
| W6R108 | BN5_4201 | Chemotaxis response regulator protein-glutamate methylesterase 1 (EC 3.1.1.61) | N | - |
| W6R8R3 | BN5_4212 | Orotate phosphoribosyltransferase (OPRT) (OPRTase) (EC 2.4.2.10) | N | - |
| W6RLS9 | BN5_4295 | Uncharacterized protein | N | - |
| W6RLW8 | BN5_4345 | Alpha/beta hydrolase fold (EC 3.-.-.-) | N | - |
| W6R1I6 | BN5_4391 | Alkyl hydroperoxide reductase AhpD (EC 1.11.1.15) | N | - |
| W6R959 | BN5_4392 | Probable tonB-dependent receptor yncD | N | - |
| W6R1N7 | BN5_4451 | Putative HTH-type transcriptional regulator ykgD | N | - |
| W6R9H7 | BN5_4507 | Ribosomal RNA small subunit methyltransferase G (EC 2.1.1.170) (16S rRNA 7-methylguanosine methyltransferase) (16S rRNA m7G methyltransferase) | N | - |
| W6RA91 | BN5_0128 | Glycolate oxidase, subunit GlcD (EC 1.1.3.15) | R | - |
| W6RAA1 | BN5_0139 | ABC transporter, solute-binding component | R | - |
| W6QS35 | BN5_0164 | D-amino acid dehydrogenase (EC 1.4.99.-) | R | - |

| | | | | |
|---|---|---|---|---|
| W6QQU2 | BN5_0273 | Acetylornithine deacetylase (EC 3.5.1.-) (EC 3.5.1.16) | R | - |
| W6QSE5 | BN5_0294 | Formate dehydrogenase, alpha subunit (EC 1.2.1.2) | R | - |
| W6RAP3 | BN5_0295 | Sulfurtransferase FdhD | R | - |
| W6QXM7 | BN5_0297 | LysM domain/BON superfamily protein | R | - |
| W6QXQ0 | BN5_0332 | N-carbamoyl-L-amino acid amidohydrolase (EC 3.5.1.87) | R | - |
| W6QSI1 | BN5_0334 | Methyl-accepting chemotaxis protein I | R | - |
| W6QST4 | BN5_0439 | ABC-type transporter periplasmic component protein | R | - |
| W6RB18 | BN5_0440 | ABC transporter inner membrane subunit protein | R | - |
| W6QQ36 | BN5_0441 | ABC transporter/ATPase component protein | R | - |
| W6QY14 | BN5_0442 | Cyanate hydratase (Cyanase) (EC 4.2.1.104) (Cyanate hydrolase) (Cyanate lyase) | R | - |
| W6QQ40 | BN5_0446 | Cobyrinic acid a,c-diamide synthase (EC 6.3.5.10) | R | - |
| W6QQA3 | BN5_0516 | ADP-dependent (S)-NAD(P)H-hydrate dehydratase (EC 4.2.1.136) (EC 5.1.99.6) (ADP-dependent NAD(P)HX dehydratase) (NAD(P)H-hydrate epimerase) (NAD(P)HX epimerase) | R | - |
| W6QY94 | BN5_0517 | Uncharacterized protein | R | - |
| W6RBC9 | BN5_0552 | Urease accessory protein UreG | R | - |
| W6QQF7 | BN5_0578 | Urease subunit alpha (EC 3.5.1.5) (Urea amidohydrolase subunit alpha) | R | - |
| W6QYH5 | BN5_0589 | ABC transporter related | R | - |
| W6QRS0 | BN5_0590 | ABC transporter related | R | - |
| W6RBH5 | BN5_0592 | Inner-membrane translocator | R | - |
| W6QS16 | BN5_0683 | Phosphoenolpyruvate-protein phosphotransferase (EC 2.7.3.9) | R | - |
| W6QQW2 | BN5_0701 | MerR family transcriptional regulator | R | - |
| W6QTN1 | BN5_0704 | Heavy metal transport/detoxification protein | R | - |
| W6RC47 | BN5_0835 | ABC transporter, ATP-binding protein (EC 3.6.3.17) | R | - |
| W6RCL0 | BN5_1022 | TRAP-type uncharacterized transport system periplasmic component-like protein | R | - |
| W6QT87 | BN5_1095 | Uncharacterized protein | R | - |
| W6RD56 | BN5_1172 | Oxidoreductase, short chain dehydrogenase/reductase family (EC 1.-.-.-) | R | - |
| W6QSC5 | BN5_1243 | Nuclear receptor binding factor related protein (EC 1.3.1.38) | R | - |
| W6RDF9 | BN5_1293 | Putative transcriptional regulator ycf27 | R | - |
| W6RDK8 | BN5_1353 | Protein ccoG | R | - |
| W6QSP1 | BN5_1354 | Nitrite/sulfite reductase, hemoprotein beta-component, ferrodoxin-like:nitrite and sulphite reductase 4Fe-4S region (EC 1.8.1.2) | R | - |
| W6R0K4 | BN5_1355 | Uncharacterized protein | R | - |
| W6R0L7 | BN5_1370 | Pirin-like protein CC_1473 | R | - |
| W6QT28 | BN5_1491 | Arginine N-succinyltransferase (EC 2.3.1.109) | R | - |
| H9N5E1 | BN5_1632 | Nitrilase (EC 3.5.5.7) | R | - |
| H9N5E3 | BN5_1633 | Radical SAM domain-containing protein (Radical SAM domain-containing proteinBiotin synthase-related enzymeRibosomal RNA large subunit methyltransferase N) (EC 2.1.1.-) | R | - |
| H9N5E4 | BN5_1634 | Acetyltransferase (GCN5-related N-acetyltransferase) | R | - |
| H9N5E5 | BN5_1635 | AIR synthase related protein domain protein (AIR synthase-like protein) | R | - |
| H9N5D8 | BN5_1637 | FAD dependent oxidoreductase (FAD-dependent oxidoreductase) | R | - |
| W6R243 | BN5_1892 | Uncharacterized HTH-type transcriptional regulator ywbI | R | - |
| W6RF13 | BN5_1895 | Uncharacterized protein | R | - |
| W6QU90 | BN5_1901 | Uncharacterized protein | R | - |
| W6R254 | BN5_1902 | Terminal oxidase subunit I (EC 1.10.3.-) | R | - |
| W6QWX6 | BN5_1904 | Phosphoserine aminotransferase (EC 2.6.1.52) (Phosphohydroxythreonine aminotransferase) (PSAT) | R | - |

| | | | | |
|---|---|---|---|---|
| W6RF21 | BN5_1905 | Histidinol-phosphate aminotransferase (EC 2.6.1.9) (Imidazole acetol-phosphate transaminase) | R | - |
| W6QU95 | BN5_1906 | Acetylornithine aminotransferase (ACOAT) (EC 2.6.1.11) | R | - |
| W6QWY1 | BN5_1909 | Methylenetetrahydrofolate reductase (EC 1.5.1.20) | R | - |
| W6RF25 | BN5_1910 | Cysteine synthase (EC 2.5.1.47) | R | - |
| W6QUB2 | BN5_1921 | Serine hydroxymethyltransferase (SHMT) (Serine methylase) (EC 2.1.2.1) | R | - |
| W6RF39 | BN5_1925 | Aliphatic nitrilase (EC 3.5.5.7) | R | - |
| W6QUS9 | BN5_2098 | CzcB family heavy metal RND efflux membrane fusion protein | R | - |
| W6R2R4 | BN5_2099 | Outer membrane efflux protein | R | - |
| W6QUU3 | BN5_2114 | Nitrate transporter periplasmic component | R | - |
| W6QW12 | BN5_2116 | Nitrate ABC transporter, ATPase subunits C and D (EC 3.6.3.31) | R | - |
| W6QXH1 | BN5_2122 | Protein kinase (EC 2.7.1.-) | R | - |
| W6RFM9 | BN5_2123 | Assimilatory nitrite reductase (Subunit) (EC 1.7.1.4) | R | - |
| W6QUV5 | BN5_2124 | Nitrite reductase (NAD(P)H) small subunit (EC 1.7.1.4) | R | - |
| W6R2U2 | BN5_2125 | Nitrate reductase (EC 1.7.99.4) | R | - |
| W6QV68 | BN5_2194 | Peptide methionine sulfoxide reductase MsrB (EC 1.8.4.12) (Peptide-methionine (R)-S-oxide reductase) | R | - |
| W6QVB9 | BN5_2244 | Transglutaminase domain-containing protein | R | - |
| W6QWF9 | BN5_2256 | Putative two-component response regulator | R | - |
| W6RG67 | BN5_2293 | UPF0502 protein BN5_2293 | R | - |
| W6QY34 | BN5_2344 | Metallo-lactamase | R | - |
| W6R3G9 | BN5_2367 | Extracellular solute-binding protein | R | - |
| W6QWV0 | BN5_2413 | GntR family transcriptional regulator | R | - |
| W6QYC5 | BN5_2449 | Cytochrome c oxidase accessory protein, cbb3-type, CcoG | R | - |
| W6R3S8 | BN5_2477 | Nitroreductase | R | - |
| W6RGP7 | BN5_2510 | RND family efflux transporter MFP subunit | R | - |
| W6RHH5 | BN5_2731 | Uncharacterized protein | R | - |
| W6QZP0 | BN5_2918 | Putative tricarboxylic transport membrane protein | R | - |
| W6R002 | BN5_3024 | Peptidase M14, carboxypeptidase A | R | - |
| W6QXM0 | BN5_3058 | FMN-dependent NADH-azoreductase (EC 1.7.-.-) (Azo-dye reductase) (FMN-dependent NADH-azo compound oxidoreductase) | R | - |
| W6QXM5 | BN5_3063 | Oxidoreductase FAD/NAD(P)-binding subunit (EC 1.18.1.2) | R | - |
| W6QXS7 | BN5_3119 | Putative quercetin 2,3-dioxygenase PA3240 (EC 1.13.11.24) | R | - |
| W6R622 | BN5_3235 | Periplasmic oligopeptide-binding protein | R | - |
| W6QZC3 | BN5_3236 | Acetamidase/formamidase (EC 3.5.1.4) | R | - |
| W6R0W2 | BN5_3333 | Carbamate kinase | R | - |
| W6RJF8 | BN5_3397 | PhoH family protein | R | - |
| W6R6I9 | BN5_3444 | Response regulator receiver modulated diguanylate cyclase (EC 3.1.1.61) | R | - |
| W6R6N0 | BN5_3489 | Alpha/beta fold family hydrolase-like protein | R | - |
| W6QZ48 | BN5_3583 | MORN repeat-containing protein | R | - |
| W6R0H4 | BN5_3660 | MotA/TolQ/ExbB proton channel | R | - |
| W6RKG4 | BN5_3716 | Type III pantothenate kinase (EC 2.7.1.33) (PanK-III) (Pantothenic acid kinase) | R | - |
| W6RKJ9 | BN5_3771 | 4-hydroxythreonine-4-phosphate dehydrogenase (EC 1.1.1.262) (4-(phosphohydroxy)-L-threonine dehydrogenase) | R | - |
| W6R7X3 | BN5_3959 | Uncharacterized protein | R | - |
| W6R822 | BN5_4009 | Polyamine-transporting ATPase (EC 3.6.3.31) | R | - |
| W6R8A0 | BN5_4096 | Phasin-like protein | R | - |
| W6R1Q3 | BN5_4128 | 1,4-beta-D-xylan xylohydrolase (EC 3.2.1.37) | R | - |

| | | | | |
|---|---|---|---|---|
| W6R8K7 | BN5_4197 | Putative glutamine synthetase (EC 6.3.1.2) | R | - |
| W6R131 | BN5_4211 | Keratin, type II cytoskeletal 75 | R | - |
| W6R257 | BN5_4248 | Glutaryl-CoA dehydrogenase (EC 1.3.8.6) | R | - |
| W6R8X4 | BN5_4292 | Uncharacterized protein | R | - |
| W6R420 | BN5_4384 | Putative oxidoreductase (EC 1.1.1.-) | R | - |
| W6R446 | BN5_4409 | Outer membrane efflux protein | R | - |

**Table S3**. Proteins differentially expressed in *P. pseudoalcaligenes* CECT5344 in response to 3-CNA using the cyanide-containing jewelry residue as control. Proteins significantly up-regulated (fold change ≥ 2) and down-regulated (fold change ≤ 2) are shown. “CNA” or “R” indicates that proteins were detected exclusively in cells grown with 3-CNA or the residue, respectively.

| Uniprot ID | Gene locus | Protein Name | Fold Change CNA/R | *p* value |
|---|---|---|---|---|
| W6QWU9 | BN5_2775 | Uncharacterized protein | 47.42 | 0.001024 |
| W6QYH5 | BN5_0589 | ABC transporter related | 8.91 | 0.000694 |
| W6QQB2 | BN5_0076 | Extracellular solute-binding protein | 6.32 | 0.018374 |
| W6QVA5 | BN5_2229 | Aminotransferase (EC 2.6.1.-) | 6.18 | 0.00042 |
| W6R989 | BN5_4427 | Bifunctional nitrilase/nitrile hydratase NIT4B (EC 3.5.5.1) | 5.63 | 4.46E-05 |
| W6RKJ4 | BN5_3761 | Extracellular solute-binding protein | 4.23 | 9.97E-05 |
| W6RJP7 | BN5_3492 | Sulfate adenylyltransferase subunit 2 (EC 2.7.7.4) (ATP-sulfurylase small subunit) (Sulfate adenylate transferase) (SAT) | 4.08 | 0.000563 |
| W6RKW1 | BN5_3967 | Chaperone protein clpB | 4.02 | 0.008259 |
| W6QRZ0 | BN5_1113 | Glycerol-3-phosphate dehydrogenase (EC 1.1.5.3) | 3.98 | 1.21E-05 |
| W6QSQ8 | BN5_0414 | Poly(3-hydroxyalkanoate) polymerase (EC 2.3.1.-) | 3.73 | 0.014692 |
| W6QUJ2 | BN5_1560 | GTP pyrophosphokinase (EC 2.7.6.5) | 3.64 | 0.008589 |
| W6QX15 | BN5_0075 | Acetylpolyamine aminohydrolase (EC 3.5.1.98) | 3.54 | 0.002772 |
| W6RLB1 | BN5_4130 | Putative tyrosine-protein kinase epsB (EC 2.7.10.-) | 3.28 | 0.002407 |
| W6QUZ6 | BN5_1206 | Pyruvate kinase (EC 2.7.1.40) | 3.27 | 0.001894 |
| W6QWS7 | BN5_2388 | 3-isopropylmalate dehydratase large subunit (EC 4.2.1.33) (Alpha-IPM isomerase) (IPMI) (Isopropylmalate isomerase) | 3.19 | 4.55E-05 |
| W6QXC5 | BN5_0151 | Flavin monoamine oxidase-related protein (EC 1.4.3.4) | 3.18 | 1.21E-05 |
| W6R3W8 | BN5_4334 | Import inner membrane translocase, subunit Tim44 | 2.89 | 0.000183 |
| W6RC60 | BN5_0855 | 50S ribosomal protein L21 | 2.85 | 0.001538 |
| W6QTJ2 | BN5_0654 | NADH dehydrogenase (EC 1.6.99.3) | 2.70 | 0.00629 |
| W6RBH5 | BN5_0592 | Inner-membrane translocator | 2.69 | 0.004146 |
| W6R5P9 | BN5_3130 | Lon protease (EC 3.4.21.53) (ATP-dependent protease La) | 2.67 | 0.000383 |
| W6R0E8 | BN5_1290 | Ribonucleoside-diphosphate reductase subunit beta (EC 1.17.4.1) | 2.63 | 0.002532 |
| W6R1F1 | BN5_4010 | Spermidine/putrescine-binding periplasmic protein 1 | 2.59 | 0.006257 |
| W6QXK5 | BN5_0267 | Polyamine-transporting ATPase (EC 3.6.3.31) | 2.56 | 0.001286 |
| W6RD85 | BN5_1207 | Universal stress protein E | 2.55 | 0.001381 |
| W6R822 | BN5_4009 | Polyamine-transporting ATPase (EC 3.6.3.31) | 2.54 | 0.001585 |
| W6QXQ0 | BN5_0332 | N-carbamoyl-L-amino acid amidohydrolase (EC 3.5.1.87) | 2.48 | 0.019617 |
| W6R0M8 | BN5_1380 | 3-ketoacyl-CoA thiolase (EC 2.3.1.16) (Acetyl-CoA acyltransferase) (Beta-ketothiolase) (Fatty acid oxidation complex subunit beta) | 2.43 | 0.013043 |
| W6QUZ9 | BN5_1686 | UDP-N-acetyl-D-mannosamine dehydrogenase (EC 1.1.1.-) | 2.42 | 0.002407 |
| W6QZK4 | BN5_3332 | Ornithine carbamoyltransferase (OTCase) (EC 2.1.3.3) | 2.42 | 0.006331 |
| W6R3I5 | BN5_2387 | 3-isopropylmalate dehydratase small subunit (EC 4.2.1.33) (Alpha-IPM isomerase) (IPMI) (Isopropylmalate isomerase) | 2.41 | 0.00618 |
| W6RFP6 | BN5_2138 | Citrate synthase | 2.38 | 8.05E-05 |
| W6QY54 | BN5_3194 | Spermidine/putrescine import ATP-binding protein potA (EC 3.6.3.31) | 2.34 | 0.001656 |
| W6QYV7 | BN5_3483 | 50S ribosomal protein L13 | 2.33 | 7.88E-05 |
| W6RKD3 | BN5_3687 | 30S ribosomal protein S17 | 2.29 | 0.008624 |
| W6R0A0 | BN5_1229 | ABC transporter, binding protein | 2.28 | 0.004159 |
| W6R2X2 | BN5_4508 | tRNA uridine 5-carboxymethylaminomethyl modification enzyme MnmG (Glucose-inhibited division protein A) | 2.25 | 0.000516 |
| W6QT13 | BN5_1030 | Uncharacterized protein | 2.25 | 0.000192 |

| | | | | |
|---|---|---|---|---|
| W6QSR7 | BN5_1379 | Fatty acid oxidation complex (EC 1.1.1.35) (EC 4.2.1.17) (EC 5.1.2.3) (EC 5.3.3.8)] | 2.24 | 3.71E-06 |
| W6QSV0 | BN5_0970 | Acetolactate synthase (EC 2.2.1.6) | 2.24 | 0.012951 |
| W6QQ48 | BN5_0456 | Ribosomal RNA large subunit methyltransferase J (EC 2.1.1.266) (23S rRNA (adenine(2030)-N6)-methyltransferase) (23S rRNA m6A2030 methyltransferase) | 2.21 | 0.001192 |
| W6R342 | BN5_4098 | Phosphate acetyl/butaryl transferase (EC 2.3.1.8) | 2.18 | 0.013414 |
| W6RHN5 | BN5_2779 | Nitrate reductase (EC 1.7.99.4) | 2.18 | 0.002548 |
| W6RHY4 | BN5_2859 | Glutamate synthase (NADPH) (EC 1.4.1.13) | 2.16 | 0.001745 |
| W6QQ64 | BN5_0001 | Chromosomal replication initiator protein DnaA | 2.09 | 0.001716 |
| W6QYA7 | BN5_2902 | Elongation factor 4 (EF-4) (EC 3.6.5.n1) (Ribosomal back-translocase LepA) | 2.09 | 0.014808 |
| W6QYJ1 | BN5_0604 | Acetyl-CoA carboxylase biotin carboxylase subunit (EC 6.4.1.2) | 2.08 | 0.000201 |
| W6R1N1 | BN5_1685 | UDP-N-acetylglucosamine 2-epimerase (EC 5.1.3.14) | 2.03 | 4.54E-07 |
| W6QVB9 | BN5_2244 | Transglutaminase domain-containing protein | 2.03 | 0.003559 |
| W6R1Q8 | BN5_4133 | Mannose-1-phosphate guanylyltransferase/mannose-6-phosphate isomerase (EC 2.7.7.22) | 2.02 | 0.001117 |
| W6QVL9 | BN5_2346 | ABC transporter, solute binding prtotein | -2.01 | 0.016581 |
| W6QVZ5 | BN5_2095 | Outer membrane porin, OprD family | -2.04 | 0.001224 |
| W6QY03 | BN5_0432 | Type IV pilus assembly protein PilQ | -2.08 | 0.005334 |
| W6R2R0 | BN5_2094 | Uncharacterized protein | -2.12 | 0.021446 |
| W6RFJ3 | BN5_2092 | Copper resistance B | -2.12 | 0.001431 |
| W6RK91 | BN5_3662 | OmpW family outer membrane protein | -2.13 | 0.003553 |
| W6QTN7 | BN5_1245 | Soluble aldose sugar dehydrogenase yliI (EC 1.1.5.-) | -2.13 | 0.001305 |
| W6QS62 | BN5_0204 | Uroporphyrinogen-III synthetase (EC 4.2.1.75) | -2.15 | 0.024321 |
| W6R1T3 | BN5_4158 | Sulfate-binding protein | -2.17 | 0.001663 |
| W6RDL1 | BN5_1358 | Probable malate:quinone oxidoreductase (EC 1.1.5.4) (MQO) (Malate dehydrogenase [quinone]) | -2.21 | 0.005648 |
| W6QXH5 | BN5_2127 | Outer membrane porin F | -2.22 | 0.002247 |
| W6R1T2 | BN5_4501 | ATP synthase subunit b (ATP synthase F(0) sector subunit b) (ATPase subunit I) (F-type ATPase subunit b) (F-ATPase subunit b) | -2.24 | 0.00461 |
| W6R3F0 | BN5_2342 | PQQ-containing dehydrogenase | -2.31 | 0.000697 |
| W6R3F4 | BN5_2347 | Cytochrome c550 | -2.48 | 0.000596 |
| W6R1C9 | BN5_4326 | D-methionine-binding lipoprotein metQ | -2.52 | 0.002685 |
| W6QSI2 | BN5_0853 | 3-ketoacyl-(Acyl-carrier-protein) reductase (EC 1.1.1.100) | -2.53 | 0.000252 |
| W6R4U4 | BN5_2831 | Ribosome-recycling factor (RRF) (Ribosome-releasing factor) | -2.69 | 0.002506 |
| W6RC58 | BN5_0850 | 10 kDa chaperonin (GroES protein) (Protein Cpn10) | -2.74 | 0.028267 |
| W6RFI0 | BN5_2077 | Uncharacterized protein | -2.89 | 0.000454 |
| W6QY43 | BN5_2354 | ABC transporter periplasmic protein | -2.98 | 0.000276 |
| W6QSL9 | BN5_0888 | OmpA/MotB domain-containing protein | -3.01 | 0.000566 |
| W6QXK4 | BN5_2157 | Outer membrane lipoprotein OprI | -3.15 | 0.001355 |
| W6QXN7 | BN5_3079 | OmpA domain-containing protein | -3.24 | 0.001928 |
| W6R788 | BN5_3674 | 30S ribosomal protein S13 | -3.65 | 0.014629 |
| W6QQS0 | BN5_0248 | Binding protein component of ABC iron transporter | -4.33 | 2.13E-05 |
| W6QS44 | BN5_1148 | Transcriptional regulator, putative | -5.21 | 0.007786 |
| W6QWM8 | BN5_1817 | Uncharacterized protein | -5.89 | 7.53E-05 |
| W6QWP5 | BN5_2353 | Uncharacterized protein | -6.59 | 0.028153 |
| W6QZ36 | BN5_2694 | 5-methyltetrahydropteroyltriglutamate--homocysteine methyltransferase (EC 2.1.1.14) (Cobalamin-independent methionine synthase) (Methionine synthase, vitamin-B12 independent isozyme) | -7.74 | 0.002967 |
| H9N5D8 | BN5_1637 | FAD dependent oxidoreductase (FAD-dependent oxidoreductase) | -11.91 | 2.08E-05 |

| | | | | |
|---|---|---|---|---|
| W6QUU3 | BN5_2114 | Nitrate transporter periplasmic component | -12.13 | 4.22E-05 |
| W6R2U2 | BN5_2125 | Nitrate reductase (EC 1.7.99.4) | -24.11 | 0.001979 |
| H9N5E2 | BN5_1631 | Uncharacterized protein | -24.63 | 7.63E-06 |
| W6RFM9 | BN5_2123 | Assimilatory nitrite reductase (Subunit) (EC 1.7.1.4) | -32.97 | 6.43E-05 |
| W6R265 | BN5_1912 | Nitrilase (EC 3.5.5.1) | -53.39 | 6.7E-08 |
| H9N5E5 | BN5_1635 | AIR synthase related protein domain protein (AIR synthase-like protein) | -70.09 | 0.000161 |
| H9N5E1 | BN5_1632 | Nitrilase (EC 3.5.5.7) | -70.61 | 8.25E-06 |
| W6RK11 | BN5_3592 | Acyl-CoA dehydrogenase family protein | -95.30 | 1.16E-06 |
| W6QYV6 | BN5_0702 | Heavy metal translocating P-type ATPase (EC 3.6.3.4) | -130.94 | 5.45E-05 |
| W6R260 | BN5_1907 | 4-hydroxy-tetrahydrodipicolinate synthase (HTPA synthase) (EC 4.3.3.7) | -139.07 | 2.44E-05 |
| W6QST4 | BN5_0439 | ABC-type transporter periplasmic component protein | -190.04 | 2.36E-05 |
| W6RF21 | BN5_1905 | Histidinol-phosphate aminotransferase (EC 2.6.1.9) (Imidazole acetol-phosphate transaminase) | -243.78 | 4.07E-06 |
| W6QUA1 | BN5_1911 | NADP-dependent malic enzyme (EC 1.1.1.40) | -322.79 | 1.76E-05 |
| W6QWV7 | BN5_0025 | Ribosomal RNA small subunit methyltransferase B (EC 2.1.1.-) | CNA | - |
| W6QRT0 | BN5_0092 | Cytochrome c oxidase subunit 2 (EC 1.9.3.1) | CNA | - |
| W6QX52 | BN5_0105 | Probable GTP-binding protein EngB | CNA | - |
| W6QQG6 | BN5_0126 | Glycolate oxidase iron-sulfur subunit | CNA | - |
| W6QQI9 | BN5_0142 | ABC transporter, ATP-binding component (EC 3.6.3.21) | CNA | - |
| W6QQK7 | BN5_0168 | Cytochrome c5-like protein | CNA | - |
| W6QPN4 | BN5_0291 | ATP-binding protein of ABC transporter | CNA | - |
| W6QXM4 | BN5_0292 | ABC permease | CNA | - |
| W6RAQ9 | BN5_0315 | Protein-glutamate methylesterase CheB (EC 3.1.1.61) | CNA | - |
| W6QQZ8 | BN5_0328 | Dihydropyrimidine dehydrogenase (EC 1.3.1.2) | CNA | - |
| W6QSH6 | BN5_0329 | Glutamate synthase (EC 1.4.1.13) | CNA | - |
| W6QXX9 | BN5_0412 | Polyhydroxyalkanoate synthase, class II (EC 2.3.1.-) | CNA | - |
| W6QRB2 | BN5_0438 | Fis family transcriptional regulator | CNA | - |
| W6QSW5 | BN5_0469 | Lipopolysaccharide heptosyltransferase I (EC 2.4.-.-) | CNA | - |
| W6QY48 | BN5_0477 | Uncharacterized protein | CNA | - |
| W6RB57 | BN5_0480 | Capsule polysaccharide export protein | CNA | - |
| W6QYC6 | BN5_0544 | Hypotetical protein | CNA | - |
| W6QQV2 | BN5_0691 | Peptide chain release factor 3 (RF-3) | CNA | - |
| W6RBV0 | BN5_0710 | tRNA/tmRNA (uracil-C(5))-methyltransferase (EC 2.1.1.-) (EC 2.1.1.35) (tRNA (uracil(54)-C(5))-methyltransferase) (tRNA(m5U54)-methyltransferase) (RUMT) (tmRNA (uracil(341)-C(5))-methyltransferase) | CNA | - |
| W6RBV5 | BN5_0715 | Oligopeptide/dipeptide ABC transporter, ATPase subunit | CNA | - |
| W6QS68 | BN5_0743 | CBS domain-containing protein | CNA | - |
| W6RBX9 | BN5_0745 | PhoH family protein | CNA | - |
| W6QR22 | BN5_0761 | Acyl-CoA dehydrogenase domain-containing protein | CNA | - |
| W6RBZ4 | BN5_0770 | Uncharacterized protein | CNA | - |
| W6QTY1 | BN5_0814 | Hydrophobe/amphiphile efflux-1 (HAE1) family protein | CNA | - |
| W6QSI8 | BN5_0858 | Glutamate 5-kinase (EC 2.7.2.11) (Gamma-glutamyl kinase) (GK) | CNA | - |
| W6QRE4 | BN5_0886 | Beta-lactamase domain-containing protein | CNA | - |
| W6QRI7 | BN5_0928 | Ribosome maturation factor RimP | CNA | - |
| W6QZK3 | BN5_1009 | Putative ATP-binding component of a transport system | CNA | - |
| W6RDB7 | BN5_1247 | Conserved virulence factor B | CNA | - |
| W6RDG8 | BN5_1303 | Methyl-accepting chemotaxis serine transducer | CNA | - |
| W6R0H3 | BN5_1320 | CAIB/BAIF family protein (EC 2.8.3.16) | CNA | - |

| | | | | |
|---|---|---|---|---|
| W6R0T3 | BN5_1432 | Glycerophosphoryl diester phosphodiesterase, putative (EC 3.1.4.46) | CNA | - |
| W6RDS2 | BN5_1435 | Lipoprotein-releasing system transmembrane protein lolC | CNA | - |
| W6QU83 | BN5_1448 | Pseudouridine synthase (EC 5.4.99.-) | CNA | - |
| W6RDT9 | BN5_1455 | Malonyl CoA-acyl carrier protein transacylase (EC 2.3.1.39) | CNA | - |
| W6QVX3 | BN5_1543 | Ribosomal protein S12 methylthiotransferase RimO (S12 MTTase) (S12 methylthiotransferase) (EC 2.8.4.4) (Ribosomal protein S12 (aspartate-C(3))-methylthiotransferase) (Ribosome maturation factor RimO) | CNA | - |
| H9N5E0 | BN5_1630 | Sigma-54-dependent transcriptional regulator (Transcriptional regulator) | CNA | - |
| W6REF7 | BN5_1648 | Phospholipase D/Transphosphatidylase (EC 2.7.8.-) | CNA | - |
| W6QTY0 | BN5_1774 | Methyl-accepting chemotaxis protein tlpB | CNA | - |
| W6QVA7 | BN5_1826 | Acyl-CoA dehydrogenase family protein | CNA | - |
| W6QU68 | BN5_1874 | Replication-associated recombination protein A | CNA | - |
| W6R238 | BN5_1886 | Response regulator in two-component regulatory system | CNA | - |
| W6QXA8 | BN5_2056 | Putative CheW protein (EC 2.7.13.3) | CNA | - |
| W6RG09 | BN5_2223 | Inner membrane ABC transporter permease protein | CNA | - |
| W6R360 | BN5_2225 | Peptide ABC transporter, putative ATP-binding protein (EC 3.6.3.25) | CNA | - |
| W6QVA8 | BN5_2234 | Amidotransferase | CNA | - |
| W6RG60 | BN5_2283 | Uncharacterized protein | CNA | - |
| W6QVK2 | BN5_2331 | FAD dependent oxidoreductase (EC 1.4.3.-) | CNA | - |
| W6QX60 | BN5_2518 | Metallo-beta-lactamase family protein (EC 3.1.2.6) | CNA | - |
| W6R4B2 | BN5_2641 | Flagellar motor switch protein FliM | CNA | - |
| W6QXK9 | BN5_2657 | Nitrogen regulation protein NR(I) | CNA | - |
| W6R4M0 | BN5_2738 | Uncharacterized protein | CNA | - |
| W6QXW7 | BN5_2749 | 2,4-dienoyl-coa reductase FADH1, putative (EC 1.3.1.34) | CNA | - |
| W6QZD5 | BN5_2808 | S-formylglutathione hydrolase (EC 3.1.2.12) | CNA | - |
| W6QY32 | BN5_2822 | Acyl-[acyl-carrier-protein]--UDP-N-acetylglucosamine O-acyltransferase (UDP-N-acetylglucosamine acyltransferase) (EC 2.3.1.129) | CNA | - |
| W6QYG7 | BN5_2962 | Penicillin amidase family protein (EC 3.5.1.11) | CNA | - |
| W6QXD1 | BN5_2965 | Nicotinate phosphoribosyltransferase (NAPRTase) (EC 6.3.4.21) | CNA | - |
| W6QZX4 | BN5_2993 | Uracil-DNA glycosylase (UDG) (EC 3.2.2.27) | CNA | - |
| W6QZY9 | BN5_3008 | 3-hydroxyacyl-CoA-acyl carrier protein transferase (EC 2.4.1.-) | CNA | - |
| W6QXJ7 | BN5_3031 | ABC transporter, ATP-binding protein (EC 3.6.3.31) | CNA | - |
| W6QYS5 | BN5_3076 | 7-cyano-7-deazaguanine synthase (EC 6.3.4.20) (7-cyano-7-carbaguanine synthase) (PreQ(0) synthase) (Queuosine biosynthesis protein QueC) | CNA | - |
| W6R097 | BN5_3122 | Probable septum site-determining protein MinC | CNA | - |
| W6QZ82 | BN5_3191 | Pyrimidine biosynthesis enzyme THI13 | CNA | - |
| W6R0K2 | BN5_3192 | 3-ketoacyl-(Acyl-carrier-protein) reductase (EC 1.1.1.100) | CNA | - |
| W6RJ07 | BN5_3193 | Dihydroorotase (EC 3.5.2.3) | CNA | - |
| W6R5Z8 | BN5_3200 | Uncharacterized protein | CNA | - |
| W6QZI3 | BN5_3307 | 3-oxoacid CoA-transferase (EC 2.8.3.5) | CNA | - |
| W6R0U1 | BN5_3308 | Butyryl-CoA:acetate CoA transferase (EC 2.8.3.5) | CNA | - |
| W6QYG6 | BN5_3315 | Beta-lactamase domain-containing protein | CNA | - |
| W6QZU5 | BN5_3430 | Riboflavin biosynthesis protein (EC 2.7.1.26) (EC 2.7.7.2) | CNA | - |
| W6QYV2 | BN5_3478 | Glutathione S-transferase domain-containing protein (EC 2.5.1.18) | CNA | - |
| W6QYY4 | BN5_3513 | Nucleotide-binding protein BN5_3513 | CNA | - |
| W6R6W3 | BN5_3564 | Lipoyl synthase (EC 2.8.1.8) (Lip-syn) (LS) (Lipoate synthase) (Lipoic acid synthase) (Sulfur insertion protein LipA) | CNA | - |
| W6R709 | BN5_3604 | Oxidoreductase FAD-binding region (EC 1.17.1.1) | CNA | - |
| W6R1P1 | BN5_3646 | Riboflavin biosynthesis protein RibD | CNA | - |

| | | | | |
|---|---|---|---|---|
| W6QZN4 | BN5_3717 | Bifunctional ligase/repressor BirA (Biotin operon repressor) (Biotin--[acetyl-CoA-carboxylase] ligase) (EC 6.3.4.15) (Biotin--protein ligase) (Biotin-[acetyl-CoA carboxylase] synthetase) | CNA | - |
| W6QZR6 | BN5_3762 | Polyamine-transporting ATPase (EC 3.6.3.31) | CNA | - |
| W6QZS5 | BN5_3772 | Ribosomal RNA small subunit methyltransferase A (EC 2.1.1.182) (16S rRNA (adenine(1518)-N(6)/adenine(1519)-N(6))-dimethyltransferase) (16S rRNA dimethyladenosine transferase) (16S rRNA dimethylase) (S-adenosylmethionine-6-N', N'-adenosyl(rRNA) dimethyltransferase) | CNA | - |
| W6R2H4 | BN5_3906 | Acyl-CoA dehydrogenase domain-containing protein (EC 1.3.99.-) | CNA | - |
| W6R1C2 | BN5_3980 | tRNA (guanine-N(7)-)-methyltransferase (EC 2.1.1.33) (tRNA (guanine(46)-N(7))-methyltransferase) (tRNA(m7G46)-methyltransferase) | CNA | - |
| W6R0C3 | BN5_4008 | UPF0064 protein yccW | CNA | - |
| W6R1M3 | BN5_4097 | Poly(3-hydroxyalkanoate) synthetase (EC 3.1.1.-) | CNA | - |
| W6R1R3 | BN5_4138 | dTDP-4-dehydrorhamnose reductase (EC 1.1.1.133) | CNA | - |
| W6R8R3 | BN5_4212 | Orotate phosphoribosyltransferase (OPRT) (OPRTase) (EC 2.4.2.10) | CNA | - |
| W6RLW8 | BN5_4345 | Alpha/beta hydrolase fold (EC 3.-.-.-) | CNA | - |
| W6R2I3 | BN5_4383 | Uncharacterized protein PYRAB12460 | CNA | - |
| W6R1I6 | BN5_4391 | Alkyl hydroperoxide reductase AhpD (EC 1.11.1.15) | CNA | - |
| W6R1K1 | BN5_4411 | Heavy metal sensor histidine kinase (EC 2.7.13.3) | CNA | - |
| W6R1N7 | BN5_4451 | Putative HTH-type transcriptional regulator ykgD | CNA | - |
| W6R9H7 | BN5_4507 | Ribosomal RNA small subunit methyltransferase G (EC 2.1.1.170) (16S rRNA 7-methylguanosine methyltransferase) (16S rRNA m7G methyltransferase) | CNA | - |
| W6QS35 | BN5_0164 | D-amino acid dehydrogenase (EC 1.4.99.-) | R | - |
| W6RAP3 | BN5_0295 | Sulfurtransferase FdhD | R | - |
| W6QXM7 | BN5_0297 | LysM domain/BON superfamily protein | R | - |
| W6RAS2 | BN5_0335 | Cytochrome c | R | - |
| W6RB18 | BN5_0440 | ABC transporter inner membrane subunit protein | R | - |
| W6QQ36 | BN5_0441 | ABC transporter/ATPase component protein | R | - |
| W6QY14 | BN5_0442 | Cyanate hydratase (Cyanase) (EC 4.2.1.104) (Cyanate hydrolase) (Cyanate lyase) | R | - |
| W6QQ40 | BN5_0446 | Cobyrinic acid a,c-diamide synthase (EC 6.3.5.10) | R | - |
| W6QQA3 | BN5_0516 | ADP-dependent (S)-NAD(P)H-hydrate dehydratase (EC 4.2.1.136) (EC 5.1.99.6) (ADP-dependent NAD(P)HX dehydratase) (NAD(P)H-hydrate epimerase) (NAD(P)HX epimerase) | R | - |
| W6QTM2 | BN5_0694 | TonB-dependent siderophore receptor | R | - |
| W6QQW2 | BN5_0701 | MerR family transcriptional regulator | R | - |
| W6QTN1 | BN5_0704 | Heavy metal transport/detoxification protein | R | - |
| W6QU71 | BN5_0909 | Protein GrpE (HSP-70 cofactor) | R | - |
| W6QT87 | BN5_1095 | Uncharacterized protein | R | - |
| W6RD56 | BN5_1172 | Oxidoreductase, short chain dehydrogenase/reductase family (EC 1.-.-.-) | R | - |
| W6QSC5 | BN5_1243 | Nuclear receptor binding factor related protein (EC 1.3.1.38) | R | - |
| W6QSN1 | BN5_1344 | Glucokinase (EC 2.7.1.2) | R | - |
| W6RDK8 | BN5_1353 | Protein ccoG | R | - |
| W6QSP1 | BN5_1354 | Nitrite/sulfite reductase, hemoprotein beta-component. ferrodoxin-like:nitrite and sulphite reductase 4Fe-4S region (EC 1.8.1.2) | R | - |
| W6R0K4 | BN5_1355 | Uncharacterized protein | R | - |
| W6R0L7 | BN5_1370 | Pirin-like protein CC_1473 | R | - |
| W6RDQ8 | BN5_1420 | Uncharacterized protein yggL | R | - |
| W6QT28 | BN5_1491 | Arginine N-succinyltransferase (EC 2.3.1.109) | R | - |
| H9N5E3 | BN5_1633 | Radical SAM domain-containing protein (Radical SAM domain-containing proteinBiotin synthase-related enzymeRibosomal RNA large subunit methyltransferase N) (EC 2.1.1.-) | R | - |

| | | | | |
|---|---|---|---|---|
| H9N5E4 | BN5_1634 | Acetyltransferase (GCN5-related N-acetyltransferase) | R | - |
| W6QWG0 | BN5_1737 | Uncharacterized protein | R | - |
| W6QWL0 | BN5_1797 | Phosphohistidine phosphatase SixA (EC 3.1.3.-) | R | - |
| W6RF01 | BN5_1878 | Putative glutathione S-transferase (EC 2.5.1.18) | R | - |
| W6R243 | BN5_1892 | Uncharacterized HTH-type transcriptional regulator ywbI | R | - |
| W6RF13 | BN5_1895 | Uncharacterized protein | R | - |
| W6RF17 | BN5_1900 | Sulfite reductase (NADPH) hemoprotein beta-component (EC 1.8.1.2) | R | - |
| W6QU90 | BN5_1901 | Uncharacterized protein | R | - |
| W6R254 | BN5_1902 | Terminal oxidase subunit I (EC 1.10.3.-) | R | - |
| W6QWX6 | BN5_1904 | Phosphoserine aminotransferase (EC 2.6.1.52) (Phosphohydroxythreonine aminotransferase) (PSAT) | R | - |
| W6QU95 | BN5_1906 | Acetylornithine aminotransferase (ACOAT) (EC 2.6.1.11) | R | - |
| W6QWY1 | BN5_1909 | Methylenetetrahydrofolate reductase (EC 1.5.1.20) | R | - |
| W6RF25 | BN5_1910 | Cysteine synthase (EC 2.5.1.47) | R | - |
| W6QUB2 | BN5_1921 | Serine hydroxymethyltransferase (SHMT) (Serine methylase) (EC 2.1.2.1) | R | - |
| W6QUS9 | BN5_2098 | CzcB family heavy metal RND efflux membrane fusion protein | R | - |
| W6QW12 | BN5_2116 | Nitrate ABC transporter. ATPase subunits C and D (EC 3.6.3.31) | R | - |
| W6QXH1 | BN5_2122 | Protein kinase (EC 2.7.1.-) | R | - |
| W6QUV5 | BN5_2124 | Nitrite reductase (NAD(P)H) small subunit (EC 1.7.1.4) | R | - |
| W6QV68 | BN5_2194 | Peptide methionine sulfoxide reductase MsrB (EC 1.8.4.12) (Peptide-methionine (R)-S-oxide reductase) | R | - |
| W6R378 | BN5_2250 | Uncharacterized protein | R | - |
| W6QY34 | BN5_2344 | Metallo-lactamase | R | - |
| W6QWQ0 | BN5_2358 | Uncharacterized protein | R | - |
| W6QW03 | BN5_2491 | Patatin-like protein | R | - |
| W6R4J6 | BN5_2697 | Solvent efflux pump periplasmic linker srpA | R | - |
| W6QZP0 | BN5_2918 | Putative tricarboxylic transport membrane protein | R | - |
| W6QXM0 | BN5_3058 | FMN-dependent NADH-azoreductase (EC 1.7.-.-) (Azo-dye reductase) (FMN-dependent NADH-azo compound oxidoreductase) | R | - |
| W6QXM5 | BN5_3063 | Oxidoreductase FAD/NAD(P)-binding subunit (EC 1.18.1.2) | R | - |
| W6RIW6 | BN5_3173 | Uncharacterized protein | R | - |
| W6R0Q7 | BN5_3267 | Iron-sulfur cluster assembly scaffold protein IscU | R | - |
| W6RJH8 | BN5_3427 | Peptidyl-prolyl cis-trans isomerase (EC 5.2.1.8) | R | - |
| W6R6N0 | BN5_3489 | Alpha/beta fold family hydrolase-like protein | R | - |
| W6R1I7 | BN5_3591 | Uncharacterized protein | R | - |
| W6QZ57 | BN5_3593 | Beta-lactamase domain-containing protein | R | - |
| W6R7C9 | BN5_3704 | 50S ribosomal protein L7/L12 | R | - |
| W6R2F9 | BN5_3891 | TRAP dicarboxylate transporter, DctP subunit | R | - |
| W6R087 | BN5_3968 | UPF0001 protein | R | - |
| W6RL44 | BN5_4057 | UPF0176 protein CPS_4798 | R | - |
| W6R8A0 | BN5_4096 | Phasin-like protein | R | - |
| W6R8K7 | BN5_4197 | Putative glutamine synthetase (EC 6.3.1.2) | R | - |
| W6R8X4 | BN5_4292 | Uncharacterized protein | R | - |
| W6R2L1 | BN5_4408 | Uncharacterized protein | R | - |
| W6R446 | BN5_4409 | Outer membrane efflux protein | R | - |
| W6RM88 | BN5_4500 | ATP synthase subunit delta (ATP synthase F(1) sector subunit delta) (F-type ATPase subunit delta) (F-ATPase subunit delta) | R | - |

**Table S4**. Oligonucleotides used in this work.

| Primer | Sequence (5' → 3')[a] | Used for |
|---|---|---|
| Nit1925FB | CCGGATCCCGCCCCGGACCTGGACCTGGAAACCACT GAAGCAAC (*Bam*HI) | *nit1* mutagenesis |
| Nit1925RH | CCAAGCTTGCGTGCGTGATCCTGCGGCGTCGAAGT (*Hin*dIII) | |
| Nit4427F1H | GTACCAAGCTTATGTCGATGGGGCTGTG (*Hin*dIII) | *nit2* mutagenesis |
| Nit4427R1B | CCCTTCAAGAGCAATGTGGATCCATGGA (*Bam*HI) | |
| 4427FCH | GGCCGCCTCTGTTCCTTATGATCCT | *nit2* mutant checking |
| 4427RCH | GGCCGACGACATCAAAGTCCAAG | |
| 1912MFB1 | TGAGGATCCTGTCTGGAGTGGCCCGCTCTAGCAA (*Bam*HI) | *nit4* mutagenesis |
| 1912MRH1 | GGCGACGAAGCTTCGAGCGGATC (*Hin*dIII) | |
| 1899F1 | CAACCGAGGAATTCATATGGCTACTCG (*Eco*RI) | *mocR* mutagenesis |
| 1899R1 | AAGCATGCTACTGGGATAGGGTCTCGATAGGC (*Sph*I) | |
| 1899F2 | AAGCATCCGGGACGGGATCTGCACTTCT (*Sph*I) | |
| 1899R2 | AAAAGCTTTACGCCTCCACATCGGACCTTCA (*Hin*dIII) | |
| cioA1 | GGCTTTCTCGGCGTGATGC | qRT-PCR |
| cioA2 | TCGTGTCCCTGCGGCGTCT | |
| q1905F | CCTCGAACTGATTTTGAAAGCGATA | |
| q1905R | GACGACAAGTGTCTCAAGAGGAA | |
| cysM3 1910 | CGGAAACACGCCACTGATCGCACT | |
| cysM3 1910 | ATCCTTGACTGAGCCTCCGGGGTT | |
| 1912A | GGAAGGGCGGTGTTTCGTGATA | |
| 1912B | GTCAAAGGTAGGGGCGACCAAG | |
| nitC1 | TGAGCGTATGGTCTGGGGGCAGGG | |
| nitC2 | CGCCGCATGAATCTGCTCGCCATC | |
| nit1F | CACGAGCGCCTGATCTGGGACGAAG | |
| nit1R | AGAAGCGCGCCAGGGGGTTGTA | |
| nit2F | TTGGCGTCCTGGGGAGCGTG | |
| nit2R | GCTGTCGGGGCGCAGTAGATCTGAA | |
| qCynSF | TGCTCGAATTGCCGGCGGAGGTCTC | |
| qCynSR | GGGTCGGTGGGCACGGCCTTG | |
| q1558F | TCAAGGGCTACAAGATGATTCTGA | |
| q1558R | CTCCTCCTTGCTGACCAGGAT | |
| q1627F | CATTTTCGCTGACAACTCCCAGTC | |
| q1627R | ATGCGGCACTTGACCGAATAG | |
| q1628F | TTTCGTACCGAAGAACCTCGAC | |
| q1628R | CAGGAGATACCGCAGAGAATGC | |
| q0412F | AAGCTCTTGTCGTTGGATAGATCG | |

| | | |
|---|---|---|
| q0412R | ACTGATCCAGTACAAACCGATGAG | |
| q2229F | AAGATGATGATTCTCGGCTTCCCTT | |
| q2229R | CTTCCAACCGTCATAAACGATGTC | |
| q1603F | GAGCATTACGGTTTGCAGTTTCTC | |
| q1603R | GTATTCACCGTCCAGTTCGTTCT | |
| rpoBF | AGCTGCTGCGTGCGATCTTCGGTGAGA | |
| rpoBR | CCAATTGCTCGTTCAGGGCGTCGTCAG | |

[a]The restriction enzyme sites indicated in brackets are underlined.

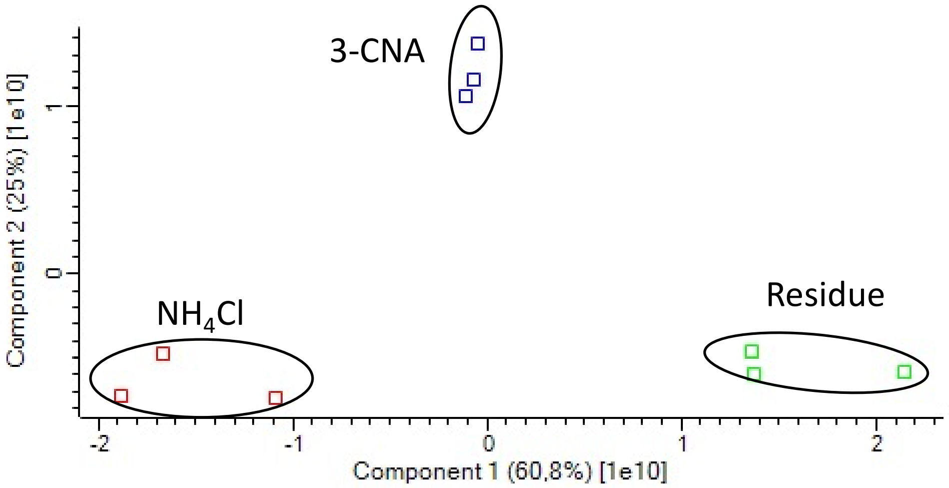


**FIG S1** Principal Component Analysis of *P. pseudoalcaligenes* CECT5344 proteomic data. Three biological replicates were used in each condition.

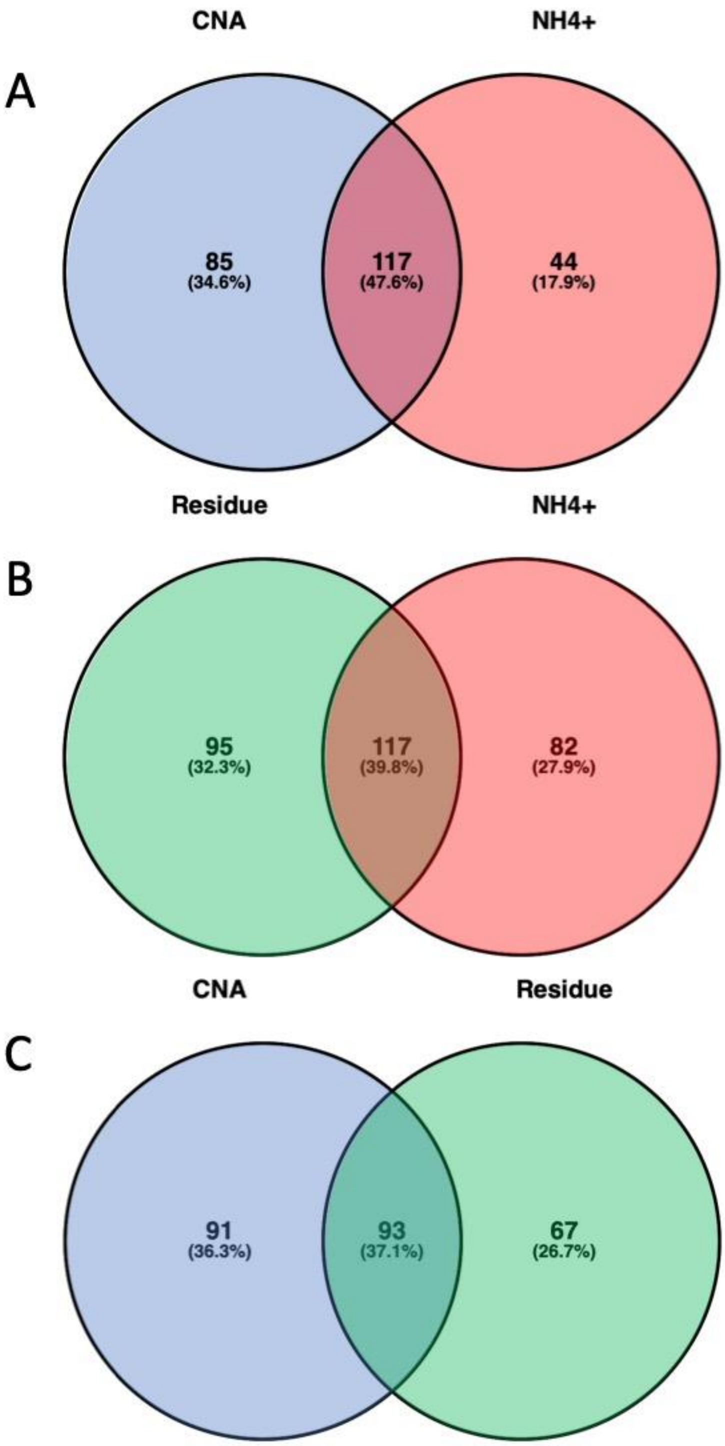


**FIG S2** Venn diagrams of *P. pseudoalcaligenes* CECT5344 proteins identified in the comparative analysis 3-CNA *vs* ammonium (A), cyanide-containing residue *vs* ammonium (B) and 3-CNA *vs* residue (C).

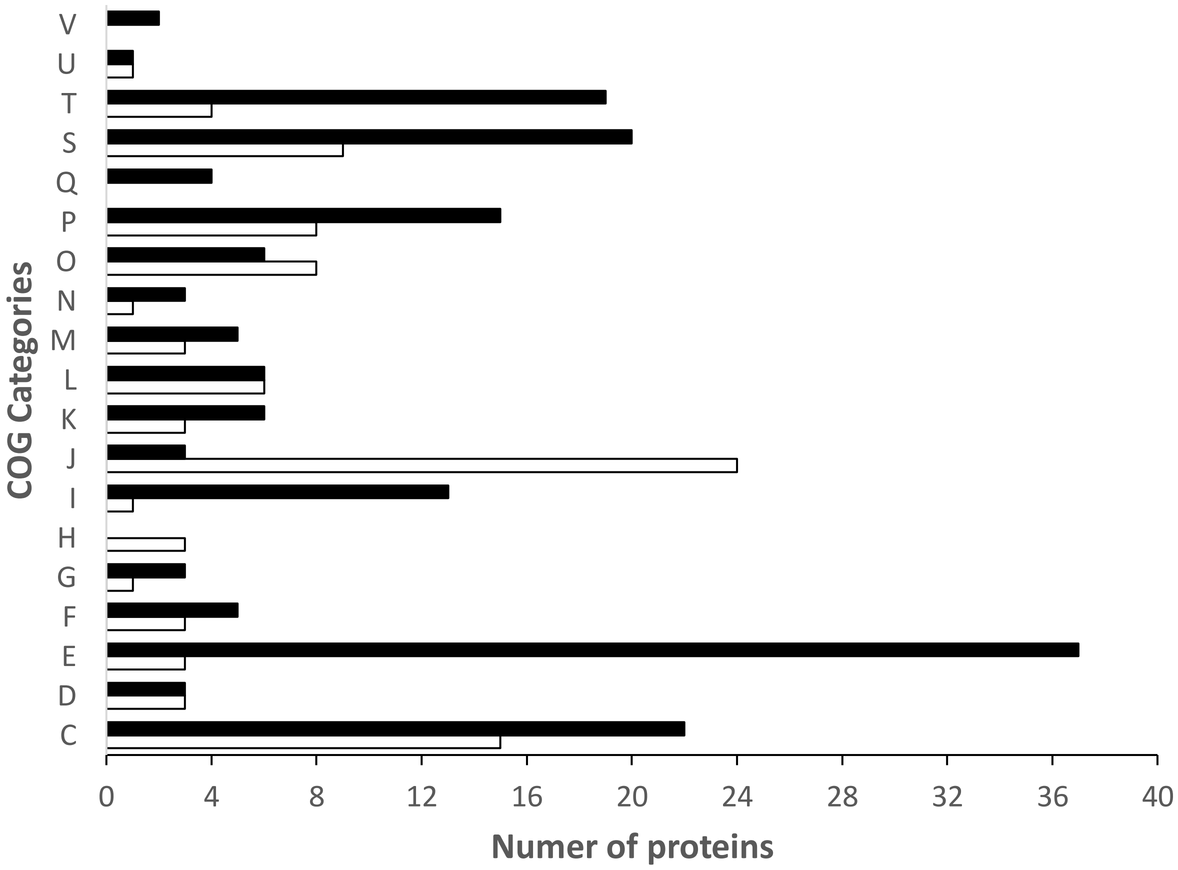


**FIG S3** Functional classification of *P. pseudoalcaligenes* CECT5344 proteins up- (black bars) or down-regulated (white bars) in response to 3-CNA using ammonium as control. The Cluster of Orthologous Genes (COG) database was used: C, energy production and conversion; D, cell cycle control, cell division, chromosome partitioning; E, amino acid transport and metabolism; F, nucleotide transport and metabolism; G, carbohydrate transport and metabolism; H, coenzyme transport and metabolism; I, lipid transport and metabolism; J, translation, ribosomal structure and biogenesis; K, transcription; L, replication, recombination and repair; M, cell wall/envelope biogenesis; N, cell motility; O, posttranslational modification, protein turnover, chaperones; P, inorganic ion transport and metabolism; Q, secondary metabolites biosynthesis, transport and catabolism; S, function unknown; T, signal transduction mechanisms; U, intracellular trafficking, secretion, and vesicular transport; V, defense mechanisms.

A

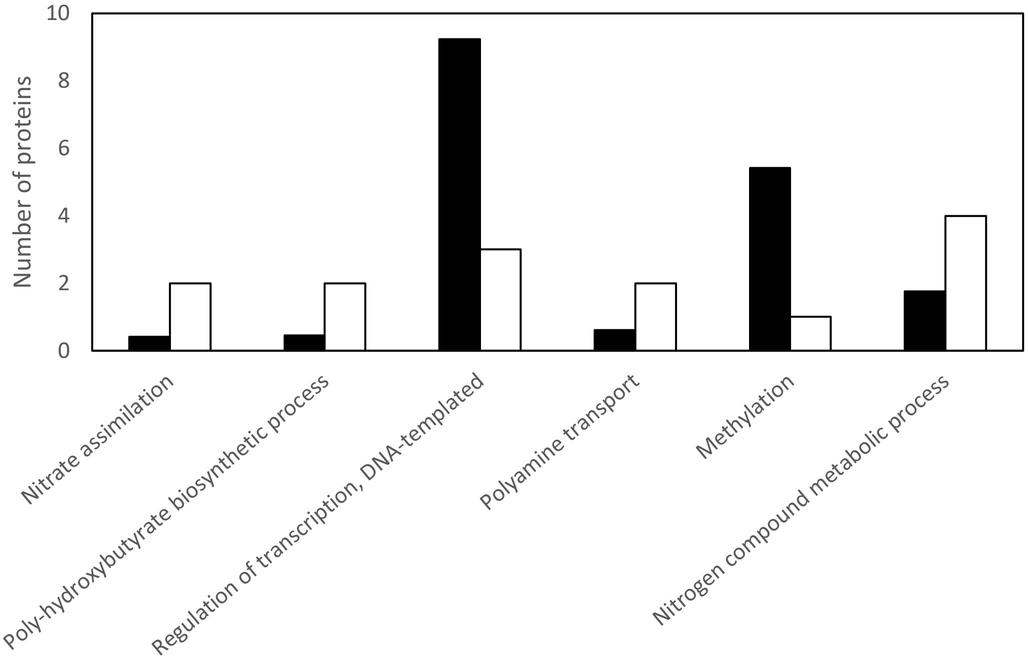


B

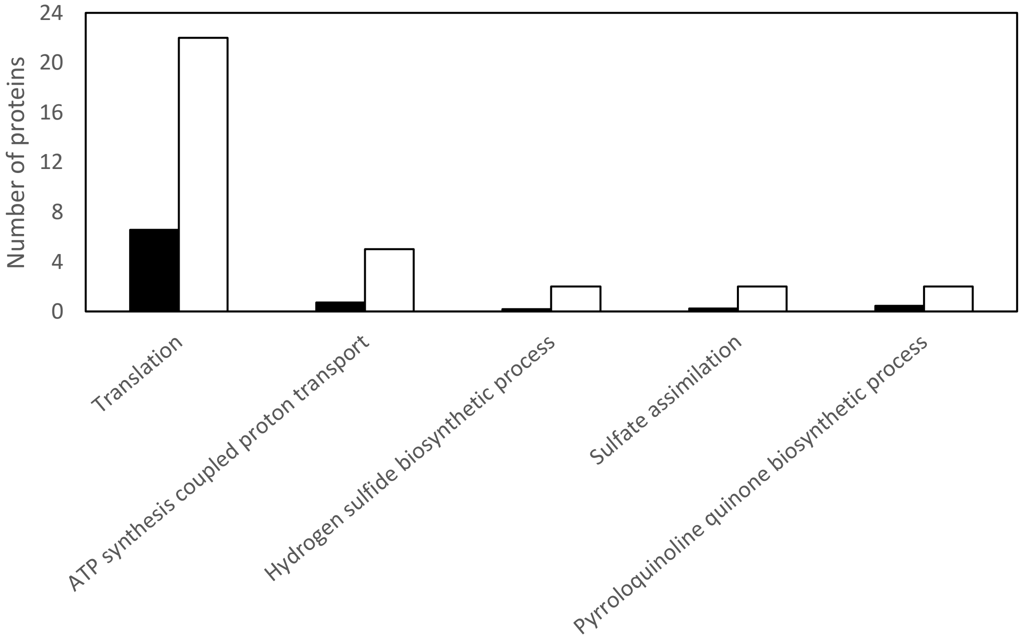


**FIG S4** Enrichment analysis of *P. pseudoalcaligenes* CECT5344 proteins differentially expressed in response to 3-CNA versus ammonium. Only significative changes (*p*-value < 0.05) in over- (A) or down-represented (B) GO terms are shown. Black bars represent the expected sample enrichment according to the GO terms distribution in the whole genome of *P. pseudoalcaligenes* CECT5344, and the actual sample enrichment obtained from the proteomic data is shown in white.

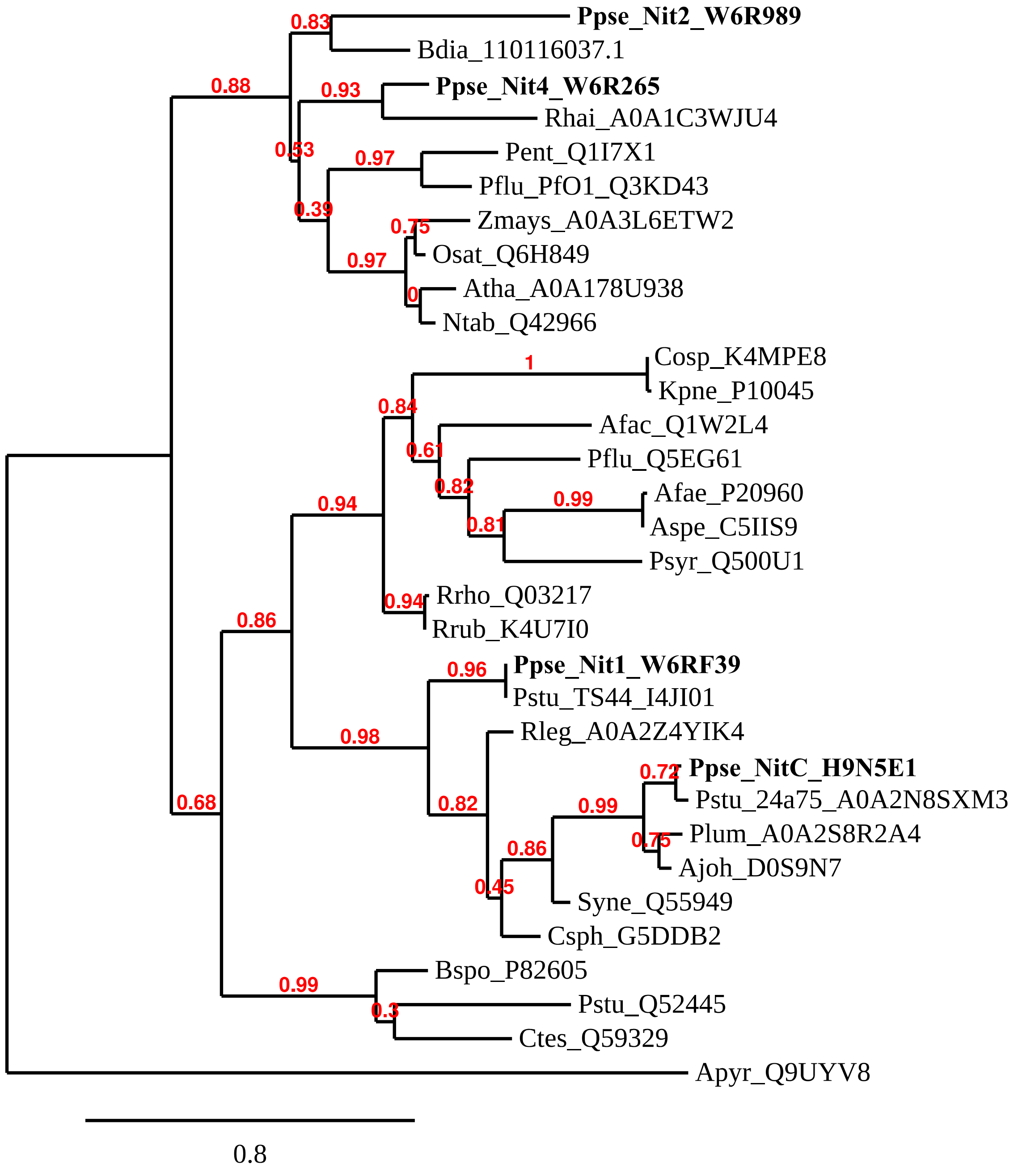


**FIG S5** Phylogenetic analysis of bacterial and plant nitrilases. The access numbers of proteins correspond to the Uniprot database. Ppse, *Pseudomonas pseudoalcaligenes* CECT5344; Pstu, *Pseudomonas stutzeri*; Rleg, *Rhizobium leguminosarum* SM144B; Rhai, *Rhizobium hainanense* CCBAU 57015; Plum, *Photorhabdus luminescens* H5; Ajoh, *Acinetobacter johnsonii* SH046; Rrho, *Rhodococcus rhodochrous* J1; Atha, *Arabidopsis thaliana*; Zmays, *Zea mays*; Psyr, *Pseudomonas syringae* pv. *syringae* B728a; Kpne, *Klebsiella pneumoniae* subs. *ozoenae*; Ntab, *Nicotiana tabacum*; Osat, *Oryza sativa*; Pent, *Pseudomonas entomophila* L48; Pflu_*Pseudomonas fluorescens* Pf0-1.
